# Supplementary material for: Multiple-trait, random regression, and compound symmetry models for analyzing multi-environment trials in maize breeding
Source: PLoS One. 2020 Nov 20;15(11):e0242705. doi: 10.1371/journal.pone.0242705 (PMC7678961; doi:10.1371/journal.pone.0242705)
Supplement: S1 Table — (DOCX) [file pone.0242705.s001.docx]

**Table S1.1. Location of the four environments (E1, E2, E3, and E4) with their respective geographic coordinates and elevations.**

| Environment | Location | Coordinates | Elevation |
| --- | --- | --- | --- |
| E1 | Jataí - Goiás | 17°55'27,63"S | 685 m |
|  |  | 51°42'45,51"W |  |
| E2 | Jataí - Goiás | 17°50'04,70"S | 865 m |
|  |  | 51°29'53,29"W |  |
| E3 | Caiapônia - Goiás | 17°30'25,06"S | 818 m |
|  |  | 51°54'35,68"W |  |
| E4 | Mineiros - Goiás | 17°23'4,72" S | 938 m |
|  |  | 52°19'39,01"W |  |
