# Supplementary material for: Multiple-trait, random regression, and compound symmetry models for analyzing multi-environment trials in maize breeding
Source: PLoS One. 2020 Nov 20;15(11):e0242705. doi: 10.1371/journal.pone.0242705 (PMC7678961; doi:10.1371/journal.pone.0242705)
Supplement: S2 Table — (DOCX) [file pone.0242705.s002.docx]

**Table S1.2. Selective accuracy for each genotype and the mean selective accuracy (below the solid line) in each environment (E1, E2, E3, and E4) based on the compound symmetry (CSM), multiple-trait (MTM) and random regression (RRM) models.**

| Genotypes | CSM | | | |  | MTM | | | |  | RRM | | | |
| --- | --- | --- | --- | --- | --- | --- | --- | --- | --- | --- | --- | --- | --- | --- |
|  | E1 | E2 | E3 | E4 |  | E1 | E2 | E3 | E4 |  | E1 | E2 | E3 | E4 |
| 1 | 0.88 | 0.88 | 0.88 | 0.88 |  | 0.89 | 0.91 | 0.82 | 0.83 |  | 0.90 | 0.90 | 0.91 | 0.80 |
| 2 | 0.88 | 0.88 | 0.88 | 0.88 |  | 0.89 | 0.91 | 0.82 | 0.83 |  | 0.90 | 0.90 | 0.91 | 0.80 |
| 3 | 0.87 | 0.87 | 0.87 | 0.87 |  | 0.86 | 0.90 | 0.82 | 0.83 |  | 0.90 | 0.89 | 0.90 | 0.80 |
| 4 | 0.88 | 0.88 | 0.88 | 0.88 |  | 0.89 | 0.91 | 0.82 | 0.83 |  | 0.90 | 0.90 | 0.91 | 0.80 |
| 5 | 0.88 | 0.88 | 0.88 | 0.88 |  | 0.89 | 0.91 | 0.82 | 0.83 |  | 0.90 | 0.90 | 0.91 | 0.80 |
| 6 | 0.88 | 0.88 | 0.88 | 0.88 |  | 0.89 | 0.91 | 0.82 | 0.83 |  | 0.90 | 0.90 | 0.91 | 0.80 |
| 7 | 0.88 | 0.88 | 0.88 | 0.88 |  | 0.89 | 0.91 | 0.82 | 0.83 |  | 0.90 | 0.90 | 0.91 | 0.80 |
| 8 | 0.88 | 0.88 | 0.88 | 0.88 |  | 0.89 | 0.91 | 0.82 | 0.83 |  | 0.90 | 0.90 | 0.91 | 0.80 |
| 9 | 0.87 | 0.88 | 0.88 | 0.88 |  | 0.89 | 0.91 | 0.82 | 0.83 |  | 0.90 | 0.90 | 0.91 | 0.80 |
| 10 | 0.88 | 0.88 | 0.88 | 0.88 |  | 0.89 | 0.91 | 0.82 | 0.83 |  | 0.90 | 0.90 | 0.91 | 0.80 |
| 11 | 0.88 | 0.88 | 0.88 | 0.88 |  | 0.89 | 0.91 | 0.82 | 0.83 |  | 0.90 | 0.90 | 0.91 | 0.80 |
| 12 | 0.88 | 0.88 | 0.88 | 0.88 |  | 0.89 | 0.91 | 0.82 | 0.83 |  | 0.90 | 0.90 | 0.91 | 0.80 |
| 13 | 0.88 | 0.88 | 0.88 | 0.88 |  | 0.89 | 0.91 | 0.82 | 0.83 |  | 0.90 | 0.90 | 0.91 | 0.80 |
| 14 | 0.88 | 0.88 | 0.88 | 0.88 |  | 0.89 | 0.91 | 0.82 | 0.83 |  | 0.90 | 0.90 | 0.91 | 0.80 |
| 15 | 0.88 | 0.88 | 0.88 | 0.88 |  | 0.89 | 0.91 | 0.82 | 0.83 |  | 0.90 | 0.90 | 0.91 | 0.80 |
| 16 | 0.88 | 0.88 | 0.88 | 0.88 |  | 0.89 | 0.91 | 0.82 | 0.83 |  | 0.90 | 0.90 | 0.91 | 0.80 |
| 17 | 0.88 | 0.88 | 0.88 | 0.88 |  | 0.89 | 0.91 | 0.82 | 0.83 |  | 0.90 | 0.90 | 0.91 | 0.80 |
| 18 | 0.88 | 0.88 | 0.88 | 0.88 |  | 0.89 | 0.91 | 0.82 | 0.83 |  | 0.90 | 0.90 | 0.91 | 0.80 |
| 19 | 0.88 | 0.88 | 0.87 | 0.87 |  | 0.89 | 0.91 | 0.82 | 0.83 |  | 0.90 | 0.90 | 0.91 | 0.80 |
| 20 | 0.88 | 0.88 | 0.88 | 0.88 |  | 0.89 | 0.91 | 0.82 | 0.83 |  | 0.90 | 0.90 | 0.91 | 0.80 |
| 21 | 0.88 | 0.88 | 0.88 | 0.88 |  | 0.89 | 0.91 | 0.82 | 0.83 |  | 0.90 | 0.90 | 0.91 | 0.80 |
| 22 | 0.88 | 0.88 | 0.88 | 0.88 |  | 0.89 | 0.91 | 0.82 | 0.83 |  | 0.90 | 0.90 | 0.91 | 0.80 |
| 23 | 0.87 | 0.87 | 0.87 | 0.87 |  | 0.89 | 0.90 | 0.81 | 0.80 |  | 0.90 | 0.90 | 0.90 | 0.77 |
| 24 | 0.88 | 0.88 | 0.88 | 0.88 |  | 0.89 | 0.91 | 0.82 | 0.83 |  | 0.90 | 0.90 | 0.91 | 0.80 |
| 25 | 0.87 | 0.87 | 0.87 | 0.87 |  | 0.89 | 0.90 | 0.81 | 0.80 |  | 0.90 | 0.90 | 0.90 | 0.77 |
| 26 | 0.88 | 0.88 | 0.88 | 0.88 |  | 0.89 | 0.91 | 0.82 | 0.83 |  | 0.90 | 0.90 | 0.91 | 0.80 |
| 27 | 0.88 | 0.88 | 0.88 | 0.88 |  | 0.89 | 0.91 | 0.82 | 0.83 |  | 0.90 | 0.90 | 0.91 | 0.80 |
| 28 | 0.88 | 0.88 | 0.88 | 0.88 |  | 0.89 | 0.91 | 0.82 | 0.83 |  | 0.90 | 0.90 | 0.91 | 0.80 |
| 29 | 0.88 | 0.88 | 0.88 | 0.88 |  | 0.89 | 0.91 | 0.82 | 0.83 |  | 0.90 | 0.90 | 0.91 | 0.80 |
| 30 | 0.88 | 0.88 | 0.88 | 0.88 |  | 0.89 | 0.91 | 0.82 | 0.83 |  | 0.90 | 0.90 | 0.91 | 0.80 |
| 31 | 0.88 | 0.88 | 0.88 | 0.88 |  | 0.89 | 0.91 | 0.82 | 0.83 |  | 0.90 | 0.90 | 0.91 | 0.80 |
| 32 | 0.88 | 0.88 | 0.88 | 0.88 |  | 0.89 | 0.91 | 0.82 | 0.83 |  | 0.90 | 0.90 | 0.91 | 0.80 |
| 33 | 0.88 | 0.88 | 0.88 | 0.88 |  | 0.89 | 0.91 | 0.82 | 0.83 |  | 0.90 | 0.90 | 0.91 | 0.80 |
| 34 | 0.88 | 0.88 | 0.88 | 0.88 |  | 0.89 | 0.91 | 0.82 | 0.83 |  | 0.90 | 0.90 | 0.91 | 0.80 |
| 35 | 0.87 | 0.87 | 0.87 | 0.87 |  | 0.86 | 0.90 | 0.82 | 0.83 |  | 0.90 | 0.89 | 0.90 | 0.80 |
| 36 | 0.88 | 0.88 | 0.88 | 0.88 |  | 0.89 | 0.91 | 0.82 | 0.83 |  | 0.90 | 0.90 | 0.91 | 0.80 |
| 37 | 0.88 | 0.88 | 0.88 | 0.88 |  | 0.89 | 0.91 | 0.82 | 0.83 |  | 0.90 | 0.90 | 0.91 | 0.80 |
| 38 | 0.88 | 0.88 | 0.88 | 0.88 |  | 0.89 | 0.91 | 0.82 | 0.83 |  | 0.90 | 0.90 | 0.91 | 0.80 |
| 39 | 0.88 | 0.88 | 0.88 | 0.88 |  | 0.89 | 0.91 | 0.82 | 0.83 |  | 0.90 | 0.90 | 0.91 | 0.80 |
| 40 | 0.88 | 0.88 | 0.88 | 0.88 |  | 0.89 | 0.91 | 0.82 | 0.83 |  | 0.90 | 0.90 | 0.91 | 0.80 |
| 41 | 0.88 | 0.88 | 0.88 | 0.88 |  | 0.89 | 0.91 | 0.82 | 0.83 |  | 0.90 | 0.90 | 0.91 | 0.80 |
| 42 | 0.88 | 0.88 | 0.88 | 0.88 |  | 0.89 | 0.91 | 0.82 | 0.83 |  | 0.90 | 0.90 | 0.91 | 0.80 |
| 43 | 0.88 | 0.88 | 0.88 | 0.88 |  | 0.89 | 0.91 | 0.82 | 0.83 |  | 0.90 | 0.90 | 0.91 | 0.80 |
| 44 | 0.88 | 0.88 | 0.88 | 0.88 |  | 0.89 | 0.91 | 0.82 | 0.83 |  | 0.90 | 0.90 | 0.91 | 0.80 |
| 45 | 0.88 | 0.88 | 0.88 | 0.88 |  | 0.89 | 0.91 | 0.82 | 0.83 |  | 0.90 | 0.90 | 0.91 | 0.80 |
| 46 | 0.88 | 0.88 | 0.88 | 0.88 |  | 0.89 | 0.91 | 0.82 | 0.83 |  | 0.90 | 0.90 | 0.91 | 0.80 |
| 47 | 0.87 | 0.87 | 0.87 | 0.87 |  | 0.89 | 0.91 | 0.80 | 0.82 |  | 0.90 | 0.89 | 0.90 | 0.79 |
| 48 | 0.88 | 0.88 | 0.88 | 0.88 |  | 0.89 | 0.91 | 0.82 | 0.83 |  | 0.90 | 0.90 | 0.91 | 0.80 |
| 49 | 0.87 | 0.87 | 0.87 | 0.87 |  | 0.86 | 0.90 | 0.82 | 0.83 |  | 0.90 | 0.89 | 0.90 | 0.80 |
| 50 | 0.88 | 0.88 | 0.88 | 0.88 |  | 0.89 | 0.91 | 0.82 | 0.83 |  | 0.90 | 0.90 | 0.91 | 0.80 |
| 51 | 0.88 | 0.88 | 0.88 | 0.88 |  | 0.89 | 0.91 | 0.82 | 0.83 |  | 0.90 | 0.90 | 0.91 | 0.80 |
| 52 | 0.88 | 0.88 | 0.88 | 0.88 |  | 0.89 | 0.91 | 0.82 | 0.83 |  | 0.90 | 0.90 | 0.91 | 0.80 |
| 53 | 0.88 | 0.87 | 0.88 | 0.88 |  | 0.89 | 0.91 | 0.82 | 0.83 |  | 0.90 | 0.90 | 0.91 | 0.80 |
| 54 | 0.88 | 0.88 | 0.88 | 0.88 |  | 0.89 | 0.91 | 0.82 | 0.83 |  | 0.90 | 0.90 | 0.91 | 0.80 |
| 55 | 0.88 | 0.88 | 0.88 | 0.88 |  | 0.89 | 0.91 | 0.82 | 0.83 |  | 0.90 | 0.90 | 0.91 | 0.80 |
| 56 | 0.88 | 0.88 | 0.88 | 0.88 |  | 0.89 | 0.91 | 0.82 | 0.83 |  | 0.90 | 0.90 | 0.91 | 0.80 |
| 57 | 0.88 | 0.88 | 0.88 | 0.88 |  | 0.89 | 0.91 | 0.82 | 0.83 |  | 0.90 | 0.90 | 0.91 | 0.80 |
| 58 | 0.87 | 0.87 | 0.87 | 0.87 |  | 0.86 | 0.90 | 0.82 | 0.83 |  | 0.90 | 0.89 | 0.90 | 0.80 |
| 59 | 0.88 | 0.88 | 0.88 | 0.87 |  | 0.89 | 0.91 | 0.82 | 0.83 |  | 0.90 | 0.90 | 0.91 | 0.80 |
| 60 | 0.88 | 0.88 | 0.88 | 0.88 |  | 0.89 | 0.91 | 0.82 | 0.83 |  | 0.90 | 0.90 | 0.91 | 0.80 |
| 61 | 0.88 | 0.88 | 0.87 | 0.88 |  | 0.89 | 0.91 | 0.82 | 0.83 |  | 0.90 | 0.90 | 0.91 | 0.80 |
| 62 | 0.88 | 0.88 | 0.88 | 0.88 |  | 0.89 | 0.91 | 0.82 | 0.83 |  | 0.90 | 0.90 | 0.91 | 0.80 |
| 63 | 0.88 | 0.88 | 0.88 | 0.88 |  | 0.89 | 0.91 | 0.82 | 0.83 |  | 0.90 | 0.90 | 0.91 | 0.80 |
| 64 | 0.88 | 0.88 | 0.88 | 0.88 |  | 0.89 | 0.91 | 0.82 | 0.83 |  | 0.90 | 0.90 | 0.91 | 0.80 |
| 65 | 0.88 | 0.88 | 0.88 | 0.88 |  | 0.89 | 0.91 | 0.82 | 0.83 |  | 0.90 | 0.90 | 0.91 | 0.80 |
| 66 | 0.88 | 0.88 | 0.88 | 0.88 |  | 0.89 | 0.91 | 0.82 | 0.83 |  | 0.90 | 0.90 | 0.91 | 0.80 |
| 67 | 0.88 | 0.88 | 0.88 | 0.88 |  | 0.89 | 0.91 | 0.82 | 0.83 |  | 0.90 | 0.90 | 0.90 | 0.77 |
| 68 | 0.87 | 0.87 | 0.87 | 0.87 |  | 0.89 | 0.91 | 0.80 | 0.82 |  | 0.90 | 0.89 | 0.90 | 0.79 |
| 69 | 0.88 | 0.88 | 0.88 | 0.88 |  | 0.89 | 0.91 | 0.82 | 0.83 |  | 0.90 | 0.90 | 0.91 | 0.80 |
| 70 | 0.88 | 0.88 | 0.88 | 0.88 |  | 0.89 | 0.91 | 0.82 | 0.83 |  | 0.90 | 0.90 | 0.91 | 0.80 |
| 71 | 0.87 | 0.87 | 0.87 | 0.87 |  | 0.89 | 0.90 | 0.81 | 0.80 |  | 0.90 | 0.90 | 0.90 | 0.77 |
| 72 | 0.88 | 0.88 | 0.88 | 0.88 |  | 0.89 | 0.91 | 0.82 | 0.83 |  | 0.90 | 0.90 | 0.91 | 0.80 |
| 73 | 0.88 | 0.88 | 0.88 | 0.88 |  | 0.89 | 0.91 | 0.82 | 0.83 |  | 0.90 | 0.90 | 0.91 | 0.80 |
| 74 | 0.88 | 0.88 | 0.88 | 0.88 |  | 0.89 | 0.91 | 0.82 | 0.83 |  | 0.90 | 0.90 | 0.91 | 0.80 |
| 75 | 0.88 | 0.88 | 0.88 | 0.88 |  | 0.89 | 0.91 | 0.82 | 0.83 |  | 0.90 | 0.90 | 0.91 | 0.80 |
| 76 | 0.88 | 0.88 | 0.88 | 0.88 |  | 0.89 | 0.91 | 0.82 | 0.83 |  | 0.90 | 0.90 | 0.91 | 0.80 |
| 77 | 0.88 | 0.88 | 0.88 | 0.88 |  | 0.89 | 0.91 | 0.82 | 0.83 |  | 0.90 | 0.90 | 0.91 | 0.80 |
| 78 | 0.87 | 0.87 | 0.87 | 0.87 |  | 0.89 | 0.91 | 0.80 | 0.82 |  | 0.90 | 0.89 | 0.90 | 0.79 |
| 79 | 0.88 | 0.88 | 0.88 | 0.88 |  | 0.89 | 0.91 | 0.82 | 0.83 |  | 0.90 | 0.90 | 0.91 | 0.80 |
| 80 | 0.88 | 0.88 | 0.88 | 0.88 |  | 0.89 | 0.91 | 0.82 | 0.83 |  | 0.90 | 0.90 | 0.91 | 0.80 |
| 81 | 0.88 | 0.88 | 0.88 | 0.88 |  | 0.89 | 0.91 | 0.82 | 0.83 |  | 0.90 | 0.90 | 0.91 | 0.80 |
| 82 | 0.88 | 0.88 | 0.88 | 0.88 |  | 0.89 | 0.91 | 0.82 | 0.83 |  | 0.90 | 0.90 | 0.91 | 0.80 |
| 83 | 0.88 | 0.88 | 0.88 | 0.88 |  | 0.89 | 0.91 | 0.82 | 0.83 |  | 0.90 | 0.90 | 0.91 | 0.80 |
| 84 | 0.88 | 0.88 | 0.88 | 0.88 |  | 0.89 | 0.91 | 0.82 | 0.83 |  | 0.90 | 0.90 | 0.91 | 0.80 |
| *mean* | 0.88 | 0.88 | 0.88 | 0.88 |  | 0.89 | 0.91 | 0.82 | 0.83 |  | 0.91 | 0.90 | 0.91 | 0.81 |
