# Supplementary material for: Multiple-trait, random regression, and compound symmetry models for analyzing multi-environment trials in maize breeding
Source: PLoS One. 2020 Nov 20;15(11):e0242705. doi: 10.1371/journal.pone.0242705 (PMC7678961; doi:10.1371/journal.pone.0242705)
Supplement: S3 Table — (DOCX) [file pone.0242705.s003.docx]

**Table S1.3.** **Genotype ranking in each environment (E1, E2, E3, and E4), based on the compound symmetry (CSM), multiple-trait (MTM), and random regression (RRM) models.**

| Genotypes | STM | | | |  | MTM | | | |  | RRM | | | |
| --- | --- | --- | --- | --- | --- | --- | --- | --- | --- | --- | --- | --- | --- | --- |
|  | E1 | E2 | E3 | E4 |  | E1 | E2 | E3 | E4 |  | E1 | E2 | E3 | E4 |
| 1 | 80 | 80 | 80 | 80 |  | 80 | 80 | 80 | 80 |  | 80 | 80 | 80 | 80 |
| 2 | 83 | 83 | 83 | 83 |  | 83 | 83 | 79 | 83 |  | 83 | 83 | 83 | 83 |
| 3 | 82 | 79 | 79 | 65 |  | 20 | 20 | 83 | 26 |  | 20 | 20 | 79 | 79 |
| 4 | 79 | 20 | 7 | 7 |  | 7 | 79 | 82 | 79 |  | 79 | 7 | 20 | 82 |
| 5 | 2 | 11 | 82 | 82 |  | 82 | 82 | 26 | 81 |  | 82 | 79 | 82 | 11 |
| 6 | 11 | 82 | 11 | 11 |  | 79 | 7 | 11 | 82 |  | 7 | 82 | 7 | 7 |
| 7 | 7 | 84 | 20 | 79 |  | 11 | 11 | 65 | 11 |  | 11 | 11 | 11 | 45 |
| 8 | 26 | 7 | 65 | 84 |  | 74 | 65 | 32 | 65 |  | 65 | 1 | 65 | 84 |
| 9 | 56 | 19 | 73 | 45 |  | 1 | 84 | 62 | 32 |  | 1 | 65 | 1 | 65 |
| 10 | 20 | 1 | 3 | 16 |  | 6 | 45 | 84 | 62 |  | 84 | 84 | 84 | 81 |
| 11 | 1 | 65 | 2 | 20 |  | 3 | 1 | 45 | 51 |  | 45 | 45 | 45 | 20 |
| 12 | 45 | 62 | 62 | 28 |  | 84 | 3 | 7 | 45 |  | 3 | 6 | 3 | 70 |
| 13 | 32 | 32 | 45 | 32 |  | 45 | 62 | 8 | 8 |  | 6 | 3 | 62 | 32 |
| 14 | 22 | 26 | 71 | 70 |  | 10 | 56 | 20 | 7 |  | 62 | 56 | 56 | 62 |
| 15 | 3 | 81 | 32 | 62 |  | 65 | 6 | 81 | 84 |  | 56 | 10 | 6 | 16 |
| 16 | 21 | 71 | 9 | 3 |  | 9 | 32 | 35 | 70 |  | 32 | 62 | 32 | 2 |
| 17 | 16 | 16 | 28 | 6 |  | 60 | 2 | 1 | 2 |  | 10 | 32 | 2 | 3 |
| 18 | 84 | 3 | 70 | 56 |  | 56 | 22 | 2 | 67 |  | 2 | 71 | 71 | 73 |
| 19 | 9 | 56 | 81 | 74 |  | 22 | 71 | 70 | 16 |  | 71 | 9 | 10 | 67 |
| 20 | 62 | 55 | 54 | 73 |  | 28 | 10 | 56 | 35 |  | 22 | 2 | 22 | 22 |
| 21 | 8 | 8 | 26 | 81 |  | 2 | 74 | 22 | 54 |  | 9 | 22 | 8 | 26 |
| 22 | 67 | 2 | 8 | 2 |  | 21 | 9 | 16 | 71 |  | 28 | 28 | 9 | 8 |
| 23 | 70 | 45 | 84 | 66 |  | 71 | 16 | 3 | 20 |  | 74 | 74 | 74 | 51 |
| 24 | 65 | 21 | 56 | 22 |  | 73 | 28 | 75 | 56 |  | 8 | 8 | 28 | 56 |
| 25 | 6 | 31 | 22 | 10 |  | 62 | 8 | 51 | 43 |  | 16 | 60 | 16 | 71 |
| 26 | 60 | 35 | 69 | 60 |  | 16 | 73 | 21 | 24 |  | 21 | 21 | 21 | 75 |
| 27 | 81 | 22 | 36 | 21 |  | 61 | 21 | 71 | 22 |  | 60 | 16 | 60 | 49 |
| 28 | 34 | 43 | 16 | 1 |  | 32 | 60 | 67 | 3 |  | 54 | 54 | 54 | 54 |
| 29 | 73 | 34 | 19 | 8 |  | 58 | 54 | 54 | 49 |  | 73 | 73 | 73 | 19 |
| 30 | 10 | 69 | 74 | 67 |  | 42 | 70 | 13 | 13 |  | 67 | 75 | 67 | 14 |
| 31 | 30 | 67 | 35 | 30 |  | 31 | 67 | 6 | 14 |  | 75 | 67 | 75 | 1 |
| 32 | 38 | 10 | 4 | 49 |  | 75 | 75 | 43 | 75 |  | 70 | 30 | 70 | 34 |
| 33 | 31 | 68 | 30 | 75 |  | 69 | 34 | 60 | 1 |  | 34 | 34 | 81 | 38 |
| 34 | 75 | 74 | 39 | 9 |  | 30 | 81 | 24 | 34 |  | 81 | 70 | 34 | 13 |
| 35 | 51 | 47 | 34 | 71 |  | 54 | 49 | 49 | 55 |  | 49 | 49 | 49 | 24 |
| 36 | 74 | 6 | 10 | 34 |  | 19 | 19 | 74 | 73 |  | 30 | 55 | 30 | 74 |
| 37 | 19 | 5 | 15 | 19 |  | 36 | 31 | 14 | 15 |  | 69 | 69 | 69 | 6 |
| 38 | 35 | 24 | 21 | 55 |  | 68 | 69 | 9 | 38 |  | 31 | 31 | 31 | 21 |
| 39 | 13 | 54 | 1 | 78 |  | 4 | 13 | 19 | 19 |  | 55 | 13 | 19 | 35 |
| 40 | 71 | 4 | 13 | 24 |  | 70 | 55 | 17 | 17 |  | 19 | 19 | 55 | 4 |
| 41 | 54 | 75 | 24 | 14 |  | 23 | 30 | 28 | 21 |  | 13 | 81 | 13 | 28 |
| 42 | 42 | 60 | 55 | 43 |  | 8 | 4 | 34 | 28 |  | 26 | 4 | 26 | 10 |
| 43 | 28 | 28 | 67 | 47 |  | 76 | 51 | 39 | 4 |  | 4 | 35 | 35 | 31 |
| 44 | 37 | 73 | 5 | 37 |  | 39 | 26 | 69 | 6 |  | 35 | 5 | 4 | 69 |
| 45 | 5 | 49 | 60 | 26 |  | 34 | 24 | 55 | 69 |  | 51 | 66 | 51 | 55 |
| 46 | 55 | 9 | 51 | 13 |  | 66 | 5 | 31 | 31 |  | 5 | 26 | 24 | 9 |
| 47 | 49 | 39 | 66 | 4 |  | 67 | 35 | 30 | 10 |  | 24 | 58 | 5 | 76 |
| 48 | 58 | 51 | 31 | 41 |  | 5 | 14 | 38 | 60 |  | 66 | 15 | 66 | 60 |
| 49 | 76 | 37 | 23 | 17 |  | 49 | 66 | 10 | 9 |  | 15 | 24 | 15 | 43 |
| 50 | 14 | 76 | 75 | 77 |  | 27 | 15 | 15 | 30 |  | 43 | 51 | 43 | 39 |
| 51 | 43 | 70 | 49 | 5 |  | 59 | 76 | 4 | 47 |  | 14 | 43 | 14 | 15 |
| 52 | 78 | 13 | 38 | 31 |  | 13 | 43 | 73 | 5 |  | 36 | 36 | 36 | 42 |
| 53 | 66 | 38 | 61 | 72 |  | 55 | 42 | 47 | 37 |  | 58 | 68 | 76 | 27 |
| 54 | 77 | 57 | 6 | 36 |  | 47 | 36 | 5 | 39 |  | 68 | 37 | 42 | 47 |
| 55 | 68 | 23 | 47 | 38 |  | 37 | 39 | 37 | 66 |  | 76 | 14 | 68 | 23 |
| 56 | 15 | 42 | 17 | 61 |  | 57 | 27 | 29 | 27 |  | 37 | 76 | 37 | 5 |
| 57 | 39 | 15 | 58 | 27 |  | 14 | 58 | 66 | 76 |  | 42 | 42 | 58 | 17 |
| 58 | 17 | 30 | 42 | 15 |  | 24 | 68 | 27 | 74 |  | 27 | 27 | 27 | 30 |
| 59 | 23 | 27 | 27 | 23 |  | 15 | 61 | 58 | 48 |  | 39 | 61 | 39 | 66 |
| 60 | 47 | 58 | 43 | 76 |  | 12 | 23 | 36 | 29 |  | 23 | 23 | 23 | 37 |
| 61 | 69 | 52 | 48 | 54 |  | 35 | 37 | 48 | 36 |  | 47 | 47 | 47 | 36 |
| 62 | 61 | 14 | 76 | 52 |  | 63 | 47 | 57 | 23 |  | 61 | 39 | 61 | 61 |
| 63 | 24 | 44 | 14 | 35 |  | 38 | 38 | 68 | 57 |  | 17 | 17 | 17 | 48 |
| 64 | 52 | 12 | 37 | 48 |  | 44 | 17 | 23 | 78 |  | 38 | 57 | 38 | 29 |
| 65 | 64 | 36 | 46 | 68 |  | 72 | 57 | 44 | 68 |  | 57 | 59 | 57 | 68 |
| 66 | 4 | 50 | 50 | 29 |  | 50 | 48 | 42 | 42 |  | 59 | 38 | 48 | 57 |
| 67 | 41 | 48 | 57 | 58 |  | 48 | 59 | 52 | 64 |  | 48 | 48 | 59 | 78 |
| 68 | 27 | 29 | 52 | 51 |  | 43 | 29 | 46 | 52 |  | 29 | 50 | 29 | 44 |
| 69 | 36 | 61 | 59 | 39 |  | 52 | 50 | 64 | 41 |  | 50 | 12 | 50 | 77 |
| 70 | 44 | 59 | 44 | 42 |  | 29 | 12 | 78 | 50 |  | 52 | 29 | 52 | 64 |
| 71 | 46 | 66 | 29 | 57 |  | 51 | 52 | 76 | 44 |  | 12 | 52 | 12 | 41 |
| 72 | 48 | 64 | 12 | 63 |  | 77 | 64 | 77 | 58 |  | 64 | 64 | 64 | 52 |
| 73 | 59 | 78 | 68 | 50 |  | 17 | 78 | 41 | 46 |  | 78 | 78 | 78 | 58 |
| 74 | 29 | 41 | 78 | 69 |  | 46 | 44 | 50 | 77 |  | 44 | 44 | 44 | 50 |
| 75 | 50 | 72 | 63 | 59 |  | 64 | 72 | 61 | 61 |  | 72 | 72 | 72 | 46 |
| 76 | 57 | 77 | 77 | 64 |  | 78 | 41 | 12 | 12 |  | 41 | 41 | 41 | 72 |
| 77 | 12 | 63 | 41 | 46 |  | 41 | 46 | 59 | 72 |  | 46 | 46 | 46 | 59 |
| 78 | 72 | 46 | 53 | 44 |  | 53 | 77 | 33 | 59 |  | 77 | 63 | 77 | 12 |
| 79 | 33 | 17 | 72 | 40 |  | 81 | 63 | 72 | 53 |  | 63 | 77 | 63 | 53 |
| 80 | 63 | 40 | 64 | 12 |  | 26 | 53 | 53 | 40 |  | 53 | 53 | 53 | 40 |
| 81 | 53 | 53 | 40 | 53 |  | 40 | 40 | 40 | 18 |  | 40 | 40 | 40 | 63 |
| 82 | 18 | 18 | 18 | 33 |  | 18 | 18 | 63 | 33 |  | 18 | 18 | 18 | 18 |
| 83 | 40 | 33 | 33 | 18 |  | 33 | 33 | 18 | 63 |  | 33 | 33 | 33 | 33 |
| 84 | 25 | 25 | 25 | 25 |  | 25 | 25 | 25 | 25 |  | 25 | 25 | 25 | 25 |
