# Supplementary material for: Multiple-trait, random regression, and compound symmetry models for analyzing multi-environment trials in maize breeding
Source: PLoS One. 2020 Nov 20;15(11):e0242705. doi: 10.1371/journal.pone.0242705 (PMC7678961; doi:10.1371/journal.pone.0242705)
Supplement: Attachment — Submitted filename: PONE-D-20-15130_R1.pdf [file pone.0242705.s005.pdf]

PLOS ONE

Multiple-trait, random regression, and compound symmetry models for analysis of multi-environment trials in maize breeding

--Manuscript Draft--

**Manuscript Number:**	PONE-D-20-15130R1
**Article Type:**	Research Article
**Full Title:**	Multiple-trait, random regression, and compound symmetry models for analysis of multi-environment trials in maize breeding
**Short Title:**	Modeling analyses of multi-environment trials in maize breeding
**Corresponding Author:**	Leonardo Bhering<br>Universidade Federal de Vicosa<br>Viçosa, MG BRAZIL
**Keywords:**	REML; BLUP; Mixed models; covariance structures; genotype-by-environment interaction; genetic selection; selective accuracy.
**Abstract:**	Modelling covariance structures is a good way to improve statistical capability to make more accurate estimations and predictions in multi-environmental trials (MET) analysis. By modelling the random terms of the statistical model, as genetic, genotype-by-environment interaction (GEI) and residual effects, there will be more accurate predictions, and the reliability in the genetic selection tends to increase. In this way, this study compared the multiple-trait (MTM), random regression (RRM), and compound symmetry (CSM) models, and their homogeneous and heterogeneous residual structures. For this, phenotypic data of 84 hybrids of maize tested in four environments were used. The goodness-of-fit were indicated for each model by Akaike information criterion (AIC). Then, we evaluated the variance components, genetic parameters, and genetic gains considering four intensities of selection (5, 10, 15 and 20 genotypes), over the four environments assessed. According to AIC, the CSM, RRM (Legendre Polynomial of order 2) and MTM with heterogeneous residual variance presented the best fits. CSM presented a unique genotypic variance and GEI variance estimate, having just the residual variances estimated per environment. Conversely, MTM and RRM were capable to estimate a genotypic variance for each environment. This characteristic carried out the MTM and RRM to higher heritability values (>0.40), while the heritabilities estimated by CSM were lower (<0.27). Similar performance was observed in selective accuracy values where CSM presented inferior values when compared with MTM and RRM for all environments, excepted for one out of four environments, what could be a super-estimated value by CSM. The selection gains followed the same pattern than the previous parameters, where CSM presented the lowest predicted gains and MTM presented the highest predicted gains, since it is the more complete model. RRM was close to MTM selection gains, achieving gains around 25%. Thus, the RRM presented better results, due to the parsimonious, and, was the best suited model to deal MET data in maize breeding.
**Order of Authors:**	Igor Ferreira Coelho
	Marco Antonio Peixoto
	Jeniffer Ferreira Santana Pinto Coelho Evangelista
	Suellen Sales
	Rodrigo Silva Alves
	Jefferson Fernando Naves Pinto
	Edesio Fialho dos Reis
	Marcos Deon Vilela de Resende
	Leonardo Bhering
**Opposed Reviewers:**	
**Response to Reviewers:**	Dear Dr. Diogo Borges Provete, we are sending the revised version of the manuscript

Powered by Editorial Manager® and ProduXion Manager® from Aries Systems Corporation

PONE-D-20-15130 - Multiple-trait, random regression, and compound symmetry models for analysis of multi-environment trials in maize breeding.
We would like to thank you and the reviewer for the excellent contributions to improvement of this manuscript. The changes, based on the questions and comments, were highlighted in blue (the same color of the answers in this letter).
Below we respond individually each comment and we are available for any questions.

Reviewer #1:
"…. Second the reviewer that this paper might have a large impact, but authors must do a much better job in accomodating the questions into the structure of the text by making it more concise and straightforward. Your main goal is relevant by itself, so avoid trying to fit in other parallel topics into the manuscript. I have provided a fully commented PDF attached. Below I listed my major concerns:"

1. Line 148: "Degrees of fit?"
Response: In the random regression analyses, the degrees of fit are given by the number of environments (or repeated measures)
2. "no need to state the formula of AIC, which is widely used. Question: why didn't you use AICc instead?" …. Table 1 (line 236) "provide deltaAICc instead of raw AIC values, which are meaningless"
Response: The AIC and BIC are the standard criteria for models choice (Cavanaugh & Neath, 2019; Neath & Cavanaugh, 2012). Models with the same fixed terms are readily compared by these criteria (Verbyla, 2019). The AIC and BIC have been widely used in model selection in plant breeding data analyses (Alves et al., 2020; Melo et al., 2020). We appreciate the suggestion, and we already cut off the AIC formula from the manuscript. We did not use the AICc because the n was large enough, 985. According to Brewer et al. (2016), the AICc is a correction in case of a small n, providing a stronger penalty than AIC for smaller sample. Although, Burnham and Anderson (2004) infers that AICc → AIC as n $\rightarrow \infty$.
3. "The abstract needs to be fully revised. It doesn't state clearly the context of the study and provide a good summary of the results and conclusions?"
We have re-written the abstract.
4. "Most part of the Results is about comparing the parameters estimated in each of the three models. Since it seems important for the paper, why don't you also provide an estimate of uncertainty around each parameter? Consider fitting models under a Bayesian framework."
As commented by the other reviewer, this article is already carrying a lot of information, in fact, would be enriching to provide an uncertainty measure about the parameter, but we believed we have enough information to discuss about the parameters. The use of Bayesian inference, with no prior information, conducts to the same results (Gianola & Fernando, 1986). In addition, the implementation of these models under Bayesian framework requires more computational efforts (processing capacity and time). The idea of fit models under Bayesian framework is very good and we will consider in a future work.
5. "The discussion must be almost entirely re-written and restructured. It barely cites other papers in the fiels, even when there're a couple of reviews on the topic. Most of paragraphs have only one or two citations, this is not good practice in scientific writing. You have to promote a dialogue between your results and previous studies."
We re-wrote and restructured the discussion as required.
6. "Most importantly, English language grammar and writing style need to be carefully revised throughout the whole manuscript. There're sentences that are really hard to understand, the reviewer also complained about it. Some parts of the text are also too much verbose, especially in the Results, try to reduce them to make it straightforward."
We made these changes. See the Results.

Thank you very much for the review and for the excellent contributions to improvement of this manuscript.

Reviewer #2:
Coelho et al present a detailed multi-environmental trial analysis of 84 maize hybrids. Their goal, as stated in the abstract, is to better understand the extent to which GEI interactions might be relevant to maize breeding programs. In order to do so, the authors use a model-fitting approach, comparing the model fit of three main model classes of widespread use (CSM, MTM and RRM). Overall, the goal of this paper is

Powered by Editorial Manager® and ProduXion Manager® from Aries Systems Corporation

clearly laid out, and the set of analyses is properly aimed at answering this key aspect of genetic variation patterns. Likewise, the amount of effort required to produce such dataset is commendable. Having said that, this version of the manuscript also has some significant shortcomings, especially with regards to organization/clarity and in placing the work in a greater context. A significant rewrite is likely required. In terms of organization and clarity, a substantive issue is the fact that the manuscript still tries to tackle too many issues at the same time, making it hard to follow. As made clear in the introduction, the goal of the manuscript is straightforward: compare these three model classes and infer the practical consequences of choosing one over the other (in terms of GEI). While the goal is clear, I feel that the manuscript falls short of such a goal, especially with regards to the interpretation of the results.

1. “While the manuscript reports detailed estimates of genetic variance, it does not address the ways in which all of these estimates have very different interpretations. For example, the estimate of GEI variance in the CSM model has no clear relationship to any of the parameters in the remaining models. However, it does have a potentially useful and clear interpretation. It can be not only interpreted as the portion of the total phenotypic variance that is due to GEI interactions, but can also be compared across traits or species, becoming therefore particularly useful. The question that emerges is then, what is the practical significance of the parameters in MTM and RRM models? To what extent can they provide a intuitive understanding of the GEI interaction?”
Response: We made the changes (see Discussion, specifically lines 326-333).
“In the random regression context, the GEI effects are detected through reaction norms (Van Eeuwijk et al., 2016). The GEI occurs when the reaction norms intersect, diverge, or converge (Van Eeuwijk et al., 2016). In the multiple-trait models, the GEI effects can not be estimated, since these effects are confounded with the genetic effects ($\hat{\sigma}_{(g_i)}^2=\hat{\sigma}_{(g_i)}^2+\hat{\sigma}_{(ge_i)}^2$). However, if the genotype ranking differs among environments, the complex GEI occurs.”

2. “Instead, right now the manuscript goes back and forth between different topics. As such, the manuscript would greatly benefit from some streamlining. The measures of selective accuracy and selection gain are, for example, rather tangential to the main goal. Selective accuracy, in particular, is purely a theoretical expectation and follows directly
from the heritability estimates. Essentially, the higher the heritability of a trait, the lower the prediction error variance. Perhaps eliminating some of these tangential aspects would help clarify the main thread. As is, the manuscript is an ensemble of variance estimates and the discussion does not help clarify their interpretation.”
Response: The selective accuracy is the most important parameter for the genetic recommendation in plant breeding (Resende & Duarte, 2007). This parameter corresponds to the correlation between predict and true genetic values (Resende & Duarte, 2007) and it is a reliability measure.

3. “I would also add that the manuscript would greatly benefit from clearer figures. Most notably, Figure 1 seems to suggest that most environments have similar rankings of maize hybrids. While some hybrids have lower or higher yield across environments, their ranking seems to remain essentially the same. Instead, the remaining of the manuscript argues for the opposite of that. Given that changes in ranking have different implications than pure increases in the yield spread, clarifying this aspect of the manuscript might be particularly important. If all the hybrids that perform well in a single environment, perform (comparatively) as well in others, then GEI interactions (while still capable of
generating variance) are of less importance for practical purposes, since all one would need to do is to select the ones with the highest yield at a single environment.”
Response: In fact, in this study, the best genotypes in one environment are also the best in the others. However, considering all genotypes in all environments the GEI is very clear, since the reaction norms cross. We cleared the figure. Similar results were found by Alves et al. (2020) evaluating Eucalyptus clones, for the Pylodin penetration trait. Besides that, these authors verified that for traits with higher coefficients of determination of GEI variances, the genotypes raking varies more along the environmental gradient.

4. “Finally, I would emphasize that ‘less is more’ in this case. The more clearly defined the manuscript becomes, the higher the impact it will have in its field.”

	"Minor comment: - The colors in most figures are hard to read and follow."<br>Response: We re-made the Results and Discuss almost entirely. (See Results and Discussion)<br><br>Thank you very much for the review and for the excellent contributions to improvement of this manuscript.<br><br><br>Alves, R. S., Resende, M. D. V., Azevedo, C. F., Silva, F. F., Rocha, J. R. do A. S. de C., Nunes, A. C. P., Carneiro, A. P. S., & Santos, G. A. (2020). Optimization of Eucalyptus breeding through random regression models allowing for reaction norms in response to environmental gradients. Tree Genetics & Genomes, 16(2), 38. https://doi.org/10.1007/s11295-020-01431-5<br>Brewer, M. J., Butler, A., & Cooksley, S. L. (2016). The relative performance of AIC, AIC C and BIC in the presence of unobserved heterogeneity. Methods in Ecology and Evolution, 7(6), 679–692. https://doi.org/10.1111/2041-210X.12541<br>Burnham, K. P., & Anderson, D. R. (2004). Multimodel inference: understanding AIC and BIC in model selection. Sociological Methods and Research, 33, 261–304.<br>Cavanaugh, J. E., & Neath, A. A. (2019). The Akaike information criterion: Background , derivation , properties , application , interpretation , and refinements. Wiley Computational Statistics, 11(January), 1–11. https://doi.org/10.1002/wics.1460<br>Gianola, D., & Fernando, R. L. (1986). Bayesian methods in animal breeding theory. Journal of Animal Science, 63(1), 217–244.<br>Melo, V. L. de, Marçal, T. de S., Rocha, J. R. A. S. de C., dos Anjos, R. S. R., Carneiro, P. C. S., & Carneiro, J. E. de S. (2020). Modeling (co)variance structures for genetic and non-genetic effects in the selection of common bean progenies. Euphytica, 216(5), 77. https://doi.org/10.1007/s10681-020-02607-9<br>Neath, A. A., & Cavanaugh, J. E. (2012). The Bayesian information criterion: background, derivation, and applications. Wiley Interdisciplinary Reviews: Computational Statistics, 4(2), 199–203. https://doi.org/10.1002/wics.199<br>Resende, M. D. V. De, & Duarte, J. B. (2007). Precisão e controle de qualidade em experimentos de avaliação de cultivares. Pesquisa Agropecuária Tropical, 37(3), 182–194. https://doi.org/10.5216/pat.v37i3.1867<br>Van Eeuwijk, F. A., Bustos-Korts, D. V., & Malosetti, M. (2016). What should students in plant breeding know about the statistical aspects of genotype × Environment interactions? Crop Science, 56(5), 2119–2140. https://doi.org/10.2135/cropsci2015.06.0375<br>Verbyla, A. P. (2019). A note on model selection using information criteria for general linear models estimated using REML. Australian and New Zealand Journal of Statistics, 61(1), 39–50. https://doi.org/10.1111/anzs.12254
**Additional Information:**	
**Question**	**Response**
**Financial Disclosure**<br><br>Enter a financial disclosure statement that describes the sources of funding for the work included in this submission. Review the submission guidelines for detailed requirements. View published research articles from *PLOS ONE* for specific examples.<br><br>This statement is required for submission and **will appear in the published article** if the submission is accepted. Please make sure it is accurate.	No, the funders had no role in study design, data collection and analysis, decision to publish, or preparation of the manuscript.

Powered by Editorial Manager® and ProduXion Manager® from Aries Systems Corporation

**Unfunded studies**<br>Enter: *The author(s) received no specific funding for this work.*<br><br>**Funded studies**<br>Enter a statement with the following details:<br>• Initials of the authors who received each award<br>• Grant numbers awarded to each author<br>• The full name of each funder<br>• URL of each funder website<br>• Did the sponsors or funders play any role in the study design, data collection and analysis, decision to publish, or preparation of the manuscript?<br>• **NO** - Include this sentence at the end of your statement: *The funders had no role in study design, data collection and analysis, decision to publish, or preparation of the manuscript.*<br>• **YES** - Specify the role(s) played.<br><br>* typeset	
**Competing Interests**<br><br>Use the instructions below to enter a competing interest statement for this submission. On behalf of all authors, disclose any competing interests that could be perceived to bias this work—acknowledging all financial support and any other relevant financial or non-financial competing interests.<br><br>This statement **will appear in the published article** if the submission is accepted. Please make sure it is accurate. View published research articles from *PLOS ONE* for specific examples.	The authors have declared that no competing interests exist.

**NO authors have competing interests**<br><br>Enter: *The authors have declared that no competing interests exist.*<br><br>**Authors with competing interests**<br><br>Enter competing interest details beginning with this statement:<br><br>*I have read the journal's policy and the authors of this manuscript have the following competing interests: [insert competing interests here]*<br><br>* typeset	
**Ethics Statement**<br><br>Enter an ethics statement for this submission. This statement is required if the study involved:<br><br>• Human participants<br>• Human specimens or tissue<br>• Vertebrate animals or cephalopods<br>• Vertebrate embryos or tissues<br>• Field research<br><br>Write "N/A" if the submission does not require an ethics statement.<br><br>General guidance is provided below. Consult the submission guidelines for detailed instructions. **Make sure that all information entered here is included in the Methods section of the manuscript.**	N/A

Powered by Editorial Manager® and ProduXion Manager® from Aries Systems Corporation

**Format for specific study types**<br><br>**Human Subject Research (involving human participants and/or tissue)**<br>• Give the name of the institutional review board or ethics committee that approved the study<br>• Include the approval number and/or a statement indicating approval of this research<br>• Indicate the form of consent obtained (written/oral) or the reason that consent was not obtained (e.g. the data were analyzed anonymously)<br><br>**Animal Research (involving vertebrate animals, embryos or tissues)**<br>• Provide the name of the Institutional Animal Care and Use Committee (IACUC) or other relevant ethics board that reviewed the study protocol, and indicate whether they approved this research or granted a formal waiver of ethical approval<br>• Include an approval number if one was obtained<br>• If the study involved *non-human primates*, add *additional details* about animal welfare and steps taken to ameliorate suffering<br>• If anesthesia, euthanasia, or any kind of animal sacrifice is part of the study, include briefly which substances and/or methods were applied<br><br>**Field Research**<br><br>Include the following details if this study involves the collection of plant, animal, or other materials from a natural setting:<br>• Field permit number<br>• Name of the institution or relevant body that granted permission	
**Data Availability**<br><br>Authors are required to make all data underlying the findings described fully available, without restriction, and from the time of publication. PLOS allows rare exceptions to address legal and ethical concerns. See the PLOS Data Policy and FAQ for detailed information.	Yes - all data are fully available without restriction

A Data Availability Statement describing where the data can be found is required at submission. Your answers to this question constitute the Data Availability Statement and **will be published in the article**, if accepted.<br><br>**Important:** Stating 'data available on request from the author' is not sufficient. If your data are only available upon request, select 'No' for the first question and explain your exceptional situation in the text box.<br><br>Do the authors confirm that all data underlying the findings described in their manuscript are fully available without restriction?	
**Describe where the data may be found in full sentences. If you are copying our sample text, replace any instances of XXX with the appropriate details.**<br><br>• If the data are **held or will be held in a public repository**, include URLs, accession numbers or DOIs. If this information will only be available after acceptance, indicate this by ticking the box below. For example: *All XXX files are available from the XXX database (accession number(s) XXX, XXX.).*<br>• If the data are all contained **within the manuscript and/or Supporting Information files**, enter the following: *All relevant data are within the manuscript and its Supporting Information files.*<br>• If neither of these applies but you are able to provide **details of access elsewhere**, with or without limitations, please do so. For example:<br><br>*Data cannot be shared publicly because of [XXX]. Data are available from the XXX Institutional Data Access / Ethics Committee (contact via XXX) for researchers who meet the criteria for access to confidential data.*<br><br>*The data underlying the results presented in the study are available from (include the name of the third party*	All relevant data are within the manuscript and its Supporting Information files

and contact information or URL).<br>• This text is appropriate if the data are owned by a third party and authors do not have permission to share the data.<br><br>* typeset	
Additional data availability information:	

Powered by Editorial Manager® and ProduXion Manager® from Aries Systems Corporation

To the Editorial Board of PLOS ONE

Dear Editor,

Please find enclosed the manuscript entitled "*Multiple-trait, random regression, and compound symmetry models for analysis of multi-environment trials in maize breeding*" for evaluation in PLOS ONE.

The authors received no specific funding for this work.

The manuscript is original, has not been published before, and is not being considered for publication elsewhere in its final form neither in printed nor in electronic format and does not present any kind of conflict of interests.

The understanding of (co)variance residual structure is an important step to find the best fitted model to your multi-environmental trial (MET) analyses. There are some different approaches to deal with genotype by environment interaction (GEI) effect, looking forward to understanding this effect and get the genotypic effect by itself as much as possible. In this way, the goal of this study was to compare some recent methodologies which deal with the GEI effect and model the residual (co)variance structure. The comparison among Compound Symetry Model (CSM), Multi-Trait Model (MTM) and Random Regression Model (RRM) was evaluated in 84 hybrids experiment over 4 different environments in the state of Goiás, Brazil, in the year 2018. The Grain Yield trait was evaluated, and the best model was found by the Akaike Information Criteria (AIC). The genetic correlation and accuracies of the methods were evaluated, as well as the ranking of the best materials. The MTM was the best fitted model to this dataset, as it presented high accuracies. The parameter numbers, which can be a negative point of this model was also reduced, because of the experimental data. Thus, this study demonstrates an important comparison among three methodologies highly adopted currently, and brings relevant results to this discussion, exposing the details (negative and positive points) of each methodology in its discussion.

Currently, Brazil is undergoing major political changes and as a result the resources available for education and research have been reduced (https://www.timeshighereducation.com/news/brazilian-scientists-protest-against-research-funding-cuts#survey-answer;https://www.nature.com/news/brazilian-scientists-reeling-as-federal-funds-slashed-by-nearly-half-1.21766). In this scenario, the Federal Universities needs to redistribute the limited resources received in an appropriate way among the various research lines to keep the postgraduate programs in operation. In this way the

availability of resources for the payment of publication fees is strictly low. Thus, I would like to request, if possible, a discount on the publication fee.

Sincerely,

Igor F. Coelho

Manuscript Click here to access/download;Manuscript;Major Revision_.docx

Multiple-trait, random regression, and compound symmetry models for analysis of multi-environment trials in maize breeding

Igor Ferreira Coelho[1] | Marco Antônio Peixoto[1] | Jeniffer Santana Pinto Coelho Evangelista[1] | Suellen Sales[1] | Rodrigo Silva Alves[1] | Jefferson Fernando Naves Pinto[2] | Edésio Fialho dos Reis[2] | Marcos Deon Vilela de Resende[3] | Leonardo Lopes Bhering[1*]

[1] Universidade Federal de Viçosa (UFV), Departamento de Biologia Geral, Viçosa, CEP: 36570-900, Minas Gerais, Brazil

[2] Universidade Federal de Jataí (UFJ), Departamento de Agronomia, Jataí, CEP: 75801-615, Goiás, Brazil

[3] Embrapa Café / Universidade Federal de Viçosa (UFV), Departamento de Estatística, Viçosa, CEP: 36570-900, Minas Gerais, Brazil

[*] Corresponding author: leonardo.bhering@ufv.br – +55 31 3612-5003

Keywords

REML; BLUP; Mixed models; covariance structures; genotype-by-environment interaction; genetic selection; selective accuracy.

Abstract

Modelling covariance structures is a good way to improve statistical capability to make more accurate estimations and predictions in multi-environmental trials (MET) analysis. By modelling the random terms of the statistical model, as genetic, genotype-by-environment interaction (GEI) and residual effects, there will be more accurate predictions, and the reliability in the genetic selection tends to increase. In this way, this study compared the multiple-trait (MTM), random regression (RRM), and compound symmetry (CSM) models, and their homogeneous and heterogeneous residual structures. For this, phenotypic data of 84 hybrids of maize tested in four environments were used. The goodness-of-fit were indicated for each model by Akaike information criterion (AIC). Then, we evaluated the variance components, genetic parameters, and genetic gains considering four intensities of selection (5, 10, 15 and 20 genotypes), over the four environments assessed. According to AIC, the CSM, RRM (Legendre Polynomial of order 2) and MTM with heterogeneous residual variance presented the best fits. CSM presented a unique genotypic variance and GEI variance estimate, having just the residual variances estimated per environment. Conversely, MTM and RRM were ~~capable to estimate~~ a genotypic variance for each environment. This characteristic carried out the MTM and RRM to higher heritability values (>0.40), while the heritabilities estimated by CSM were lower (<0.27). Similar performance was observed in selective accuracy values where CSM presented inferior values when compared with MTM and RRM for all environments, excepted for one out of four environments, what could be a ~~super-estimated value~~ by CSM. The selection gains followed the same pattern than the previous parameters, where CSM presented the lowest predicted gains and MTM presented the highest predicted gains, since it is the more complete model. RRM was close to MTM

selection gains, achieving gains around 25%. Thus, the RRM presented better results, due to the parsimonious, and, was the best suited model to deal MET data in maize breeding.

Introduction

Maize (*Zea mays* L.) is the most cultivated crop worldwide, with world yield of 1.1 billion of tons in the 2018/2019 crop year [1]. An important advantage of this crop is the capacity of being cultivated in a range of environments and different seasons. Such aspects lead to differential response by genotypes to different environments, which is known as genotype-by-environment interaction (GEI) [2], or even as phenotypic plasticity [3].

Due to environmental effects on quantitative traits, performance tests of potential genotypes are conducted in multiple years and locations. The variation in performance depends on the magnitude of GEI, which occurs when there is variation in the genotype relative performance of the genotype across the environments tested [4]. An “environment” can be defined by biotic or abiotic aspects to which the plants are exposed. There are also other characteristics used to define environments, such as technology level or plant population density [5].

The compound symmetry model (CSM) is the basic model, while the multiple-trait model (MTM) is the most complete to deal with GEI. Although the CSM is a simple and parsimonious structure, having a small number of parameters to be estimated this model have limited assumptions, such as correlation equal to 1 and unique genetic variance [6]. Moreover, MTM (unstructured model) tends to present problems regarding convergence, due to the high number of parameters to be estimated [7–9]. Then, the MTM became prohibitive when the number of environments is high [8].

In this sense, the performance of the random regression model (RRM) is similar to the MTM, whereas estimating less parameters [10]. RRM captures the continuous changing of a trait, over time or environmental gradient, using fewer parameters than MTM [9,11]. It is a classical quantitative model extension, which predicts the genetic (additive, dominance, and epistasis) component of reaction norms. The prediction of genotypic values can be also made for untested environments which fit into the environmental gradient [6]. RRM is suitable to fit complex reaction norms [12,13]. The reaction norms considers the GEI and can identify its underlying causes, being plotted over the environmental gradient (X axis) [14]. Next, these reaction norms fitted using RRM assign the predicted genotypic effect by using the random regression coefficients to build a function per genotype, according to the environmental gradient [8]. Then, there will be a genotypic value for each environment, considering GEI and the genetic covariances and residual variance changes over the environmental gradient.

Selective accuracy is the most suitable alternative to compare different statistical methods in the genetic improvement [15]. Moreover, selection gain and heritability are also used to compare methodologies which deal with GEI, by evaluating the variance components and genotypic values [5]. Maximizing the selection gain is an important goal of any breeding program. The selection gain predicts the performance that these genetic materials will provide for the trait in the following cycle (with recombination) of breeding. Accuracy gives the certainty of choosing the right progenies. Besides accuracy, it is necessary to understand the genetic basis, selection units, and heritability of a trait. It is applied for some breeding strategies, such as recurrent selection methods, for decision making in breeding programs [5].

Considering the expansion of maize crop cultivation, studies on GEI are necessary to evaluate different models dealing with this effect and indicate the most suitable to

handle with MET data to maximize the selective accuracy and the gains with selection [9]. Additionally, RRM studies have been developed to evaluate plasticity and GEI in animals and plant science, providing satisfactory results [6,16–19]. Thus, this study aims: (i) evaluate and compare three models for MET analysis: CSM, MTM, and RRM; (ii) determine which model is the best suited to estimate variance components and selective accuracies, and predict genotypic values and selection gains.

Material and Methods

Experimental data

The trials were carried out between January and July 2018, in four sites, in Goiás, Brazil (Table S1.1). The environments were considered as each evaluated site, totaling four environments (hereafter E1, E2, E3, and E4). The region presents wet temperate, with dry winters and hot summers (Cwa) [20]. Average annual temperature is around 21.5°C and average rainfall is between 1.400 and 2.000 mm year^{-1}. The agricultural practices were based on those used for maize cultivation in Brazil [21].

A data set with 78 inter-population hybrids (Table S1.2) and the six commercial hybrids (F_1) most used in the region was used. The inter-population hybrids were developed from 13 F_2 populations, derived from commercial hybrids widely adopted in the region. The trials were conducted in complete block design with three replications and 44 plants per plot. Plots consisted of four 4 m rows, with a spacing of 0.40 m between plants and 0.45 m between rows. To eliminate the competition effect, only the two central rows were evaluated. The grain yield (GY) trait was evaluated by the weight of the plot and converted to hectares (kg ha^{-1}).

Statistical analyses

Three statistical models were fitted: (i) Compound symmetry model (CSM), (ii) Multiple-trait model (MTM), and (iii) Random regression models (RRM) fitted through Legendre polynomials.

The estimation of variance components and prediction of the genotypic values for GY trait were made via restricted maximum likelihood/best linear unbiased prediction (REML/BLUP) procedure [22].

i - Compound symmetry model

The CSM was given by the following equation:

$$y = \mathbf{X}r + \mathbf{Z}g + \mathbf{W}ge + e,$$

where *y* is the vector of phenotypes; *r* is the vector of block-environment combinations (assumed to be fixed), which comprises the effects of environment and block within environment, added to the overall mean; *g* is the vector genotypic effects (assumed as random), *ge* is the vector GEI effects (random), and *e* is the vector of residuals (random). Uppercase bold letters refer to the incidence matrices for those effects.

In this model: $g \sim N(0, I\sigma_g^2)$, $ge \sim N(0, I\sigma_{ge}^2)$, and $e \sim N(0, R)$; where σ_g^2 is the genotypic variance between genotypes, σ_{ge}^2 is the GEI variance, *I* is an identity matrix with appropriate order to the respective effect, and R refers to a diagonal matrix of residual variances. Models accounting for different residual variance structures (homogeneous and heterogeneous) were tested here.

ii - Multiple-trait model

The MTM was given by the following equation:

$$y = \mathbf{X}r + \mathbf{Z}g + e.$$

In this model: $g \sim N(0, \Sigma_g \otimes I)$, and $e \sim N(0, R)$; where Σ_g is the genotypic covariance matrix, I is an identity matrix with appropriate order, $\otimes$ denotes the Kronecker product, and R refers to a diagonal matrix of residual variances. Models accounting for different residual variance structures (homogeneous and heterogeneous) were tested here.

iii - Random regression models

In order to use Legendre polynomials, the phenotypic mean of each environment (μ_i) must be scaled to range from -1 to +1. The environmental gradient values (E_i) were obtained by the following expression [23]:

$$E_i = -1 + 2[(\mu_i - \mu_{min})/(\mu_{max} - \mu_{min})].$$

The RRM were fitted through Legendre polynomials, considering all possible degrees of fit, as follows:

$$Y_{ijk} = \mu + S_j + R(S_{jk}) + \sum_{d=0}^{D} \alpha_{id} \Phi_{ijd} + e_{ijk},$$

where Y_{ijk} is the ith genotype (i = 1, 2, …, 84) in the jth environment (j = 1, 2, 3, 4) in the kth block (k = 1, 2, 3); μ is the overall mean; S_j is the fixed effect of environment j; $R(S_{jk})$ is the fixed effect of block k within environment j; d is the polynomial degree, ranging from *0* to D (D = *number of environments* - *1*); α_{id} is the random regression coefficient for the orthogonal polynomial for the genotype effects; Φ_{ijd} is the dth orthogonal polynomial for the jth environment for the ith genotype; and e_{ijk} is the residual random effect associated with Y_{ijk}.

In the matrix notation, the above model was described by the following equation:

$$y = \boldsymbol{X}r + \boldsymbol{Z}g + e.$$

In this model: $g \sim N(0, K_g \otimes I_{84})$, and $e \sim N(0, R)$; where K_g is the covariance matrix for the coefficients of genotypic effects, $\otimes$ denotes the Kronecker product, I_{84} is an identity matrix of order *84*, and R refers to a diagonal matrix of residual variances.

Models accounting for different residual variance structures (homogeneous and heterogeneous) were tested here.

Model selection

In order to compare the residual variance structures in CSM, MTM, and RRM the Akaike information criterion (AIC) [24] was used. Significance of the random effects of the three models (CSM, MTM, and RRM) were tested by the likelihood ratio test (LRT) for [25].

Variance components and genetic parameters

For CSM, phenotypic variance ($\hat{\sigma}_p^2$), broad-sense heritability (h^2), coefficient of determination of GEI (c_{ge}^2), and genotypic correlation among environments (r_{gloc}), were estimated using the following equations:

$$\hat{\sigma}_p^2 = \hat{\sigma}_g^2 + \hat{\sigma}_{ge}^2 + \hat{\sigma}_e^2,$$

$$h^2 = \frac{\hat{\sigma}_g^2}{\hat{\sigma}_p^2},$$

$$c_{ge}^2 = \frac{\hat{\sigma}_{ge}^2}{\hat{\sigma}_p^2}, \text{ and}$$

$$r_{gloc} = \frac{\hat{\sigma}_g^2}{\hat{\sigma}_g^2 + \hat{\sigma}_{ge}^2}.$$

For RRM, the estimates of the genotypic variance ($\hat{\sigma}_g^2$) and predicted genotypic values ($\tilde{g}_{ij}$), in the original scale, were estimated/predicted by the following expressions [23,26]:

$$\hat{\sigma}_g^2 = \Phi_{ijd}\hat{K}_g\Phi'_{ijd}, \text{ and}$$

$$\tilde{g}_{ij} = \sum_{d=0}^{D} \hat{\alpha}_{id}\Phi'_{ijd}.$$

For MTM and RRM, phenotypic variance ($\hat{\sigma}_p^2$) and broad-sense individual heritability (h^2) were estimated by the following expressions [8]:

$$\hat{\sigma}_p^2 = \hat{\sigma}_g^2 + \hat{\sigma}_e^2, \text{ and}$$

$$h^2 = \frac{\hat{\sigma}_g^2}{\hat{\sigma}_p^2}.$$

The selective accuracy ($r_{\hat{g}g}$) was calculated, for CSM, MTM and RRM, by the following expressions, respectively:

$$r_{\hat{g}g} = \sqrt{1 - \frac{\left[1-\left(1-\frac{PEV}{\hat{\sigma}_g^2}\right)\right]\left[1-\left(1-\frac{PEV}{\hat{\sigma}_{ge}^2}\right)\right]}{1-(1-\frac{PEV}{\hat{\sigma}_g^2})(1-\frac{PEV}{\hat{\sigma}_{ge}^2})}},$$

$$r_{\hat{g}g} = \sqrt{1 - \frac{PEV}{\hat{\sigma}_g^2}}, \text{ and}$$

$$r_{\hat{g}g} = \sqrt{1 - \frac{\Phi_{ijd} PEV \Phi'_{ijd}}{\hat{\sigma}_g^2}},$$

where PEV is the prediction error variance, obtained by the diagonal elements of the inverse of the coefficient matrix (information matrix) of the mixed model equations.

The experimental coefficient of variation (CV_e) was calculated using the $\hat{\sigma}_e$ of each environment:

$$CV_e = \frac{\hat{\sigma}_e}{\mu},$$

where μ is the phenotypic mean of the experiment.

Selection gain

Rankings per environment were carried out for each method. Then, selection gain (*SG*) was predicted, for each environment, considering the selection of 20, 15, 10 and 5 genotypes, based on the following expression [27]:

$$SG\ (\%) = \left(\frac{[(\Sigma_{i=1}^{n} GV_i)/n] - \mu_p}{\mu_p}\right) x\ 100,$$

where: GV_i is the predicted genetic value of genotype i, n is the number of genotypes selected, and μ_p is the overall mean of the population.

Software

All analyses were conducted in the ASReml 4.1 [22] and ASReml-R, implemented in the R software [28,29].

Results

Model selection and significance of the random effects

Based on AIC, the selected compound symmetry model (CSM) was *CS.Rhe*. For MTM, the best one was *MT.Rhe,* and, among the RRM, the best one was *RR.2.Rhe* (Table 1). Hence, these three models, were used to estimate the variance components and to predict the genotypic values. Considering all models of this study, the RRM was a best fitted model comparing to MTM and CSM, as stated by AIC. In addition, according to the LRT, significant genotype effects were detected by all models (CSM, MTM, and RRM) and GEI effects was detected by CSM (Table 1).

Table 1. Model fitted, Akaike information criterion (AIC), and the likelihood ratio test (LRT) for genotypic and GEI effects, between parentheses, for the GY trait assessed in 84 interpopulational hybrids evaluated in four environments

Model[a]	k[b]	AIC	LRT
CS.Rho	3	14904.06	72.16** (14.11**)
CS.Rhe	6	14895.57[#]	74.06** (11.92**)
MT.Rho	11	14888.15	211.74**
MT.Rhe	14	14881.71[#]	203.61**
RR.1.Rho	2	14764.32	82.95**
RR.2.Rho	4	14751.88	91.17**
RR.3.Rho	7	14754.96	92.63**
RR.4.Rho	11	14736.76	105.73**

RR.1.Rhe	5	14753.98	81.08**
RR.2.Rhe	7	14738.36[#]	90.89**
RR.3.Rhe	10	14741.68	92.23**
RR.4.Rhe	14	14748.26	92.94**

[a] Compound symmetry models were referred to as *CS.R_*, multiple-trait models were referred to as *MT.R_*, and random regression models were referred as *RR.O.R_*; where *R_* may assumed homogeneous (Rho) or heterogenous (Rhe) residual variance structures, and *O* represents the orthogonal polynomial order fitted to the genotypic effect. [b] Number of parameters; [#]indicated the selected model by AIC (lower AIC); ** Significant at 1% of probability by the chi-square test. The null hypothesis tested was that random effects did not differ from zero.

Variance components and genetic parameters

Single estimates of genotypic and GEI variances were given by CSM (Table 2). Conversely, MTM and RRM presented one estimative of genetic variances for each environment but was not obtained any GEI variance estimation (Table 2). Regarding to MTM and RRM, specifically, the highest values, in both models, of genotypic variance ($\hat{\sigma}_g^2$) were detected in environments E1 and E2, respectively. The environment E4 presented the lowest values for both models, while the environment E3 presented the highest values of residual variance ($\hat{\sigma}_e^2$) (Table 2).

Table 2. Variance components and genetic parameters by the three models: compound symmetry (CSM), multiple-trait (MTM), and random regression (RRM).

Component/Parameter	CSM	MTM	RRM
$\hat{\sigma}_{g1}^2$	324884.60	785802.30	467421.77
$\hat{\sigma}_{g2}^2$		537714.80	533106.91

$\hat{\sigma}^2_{g3}$		313339.20	391987.53
$\hat{\sigma}^2_{g4}$		250269.40	226533.74
$\hat{\sigma}^2_{e1}$	971531.00	852670.90	1054350.00
$\hat{\sigma}^2_{e2}$	743312.90	752053.70	753868.00
$\hat{\sigma}^2_{e3}$	1092919.30	1125631.50	1208480.00
$\hat{\sigma}^2_{e4}$	678742.60	690943.60	702445.00
$\hat{\sigma}^2_{ge}$	124696.50	-	-
h^2_1	0.23	0.48	0.31
h^2_2	0.27	0.42	0.41
h^2_3	0.21	0.22	0.24
h^2_4	0.29	0.27	0.24
c^2_1	0.11	-	-
c^2_2	0.14	-	-
c^2_3	0.10	-	-
c^2_4	0.16	-	-
$r_{\hat{g}g_1}$	0.88	0.89	0.91
$r_{\hat{g}g_2}$		0.91	0.90
$r_{\hat{g}g_3}$		0.82	0.91
$r_{\hat{g}g_4}$		0.83	0.81
CV_{e1}	13.04	12.21	13.58
CV_{e2}	11.01	11.07	11.09
CV_{e3}	14.53	14.74	15.28
CV_{e4}	14.74	14.87	14.99
r_{gloc}	0.72	-	-

μ_1	7560.18
μ_2	7832.41
μ_3	7196.73
μ_4	5590.70

$\hat{\sigma}^2_{gi}$: genotypic variance in the environment *i*; $\hat{\sigma}^2_{ei}$: residual variance in the environment *i*; $\hat{\sigma}^2_{ge}$: genotype-by-environment interaction variance; h^2_i: heritability in the environment *i*; c^2_i: coefficient of determination of genotype-by-environment effect in the environment *i*; $r_{\hat{g}g_i}$: selective accuracy in environment *i*; CV_{ei}: experimental coefficient of variation in the environment *i*; r_{gloc}: genotypic correlation among environments; μ_i: phenotypic mean for the GY trait in the environment *i*.

Heritabilities (h^2) estimates did not follow any pattern. By CSM the estimates of heritabilities varied from 0.21 (E3) to 0.29 (E4) (Table 2). By the MTM and RRM similar results were obtained (Table 2). Regarding selective accuracy ($r_{\hat{g}g}$), the CSM estimated a single value for all environments (0.88), while MTM and RRM estimated selective accuracies for each environment (Tables 2 and S1.3). The environment E4 was the only one where CSM presented higher selective accuracy comparing to the other models (Tables 2 and S1.3). On the other environments, the selective accuracies of MTM or RRM were slightly superior over the CSM (Tables 2 and S1.3). For CSM, the genotypic correlation performance among environments (r_{gloc}) was 0.72.

Reaction norms

In Fig 1, the reaction norms were adjusted through Legendre polynomials of degree 2, which confirms the presence of the GEI effect, as the ranking changes over the environmental gradient (Table S1.4) [30]. When the genotypes vary their performances

over the environments, it is a complex interaction, since the reaction norms cross and ranking changes among the four environments.

Fig 1: Reaction norms of the 84 genotypes. The red lines refer to the five best and worst genotypes performances over the environmental gradient.

Selection gain

The highest predict gains were observed in environments E1 and E2, for both three models (Table 3). MTM obtained the highest gains, in all scenarios, being five selected genotypes, in environment E1, the highest gain overall (25.54%). As expected, gain decreases as the number of selected genotypes increases, i.e., the scenario of 20 selected genotypes, by CSM, predicted gain of 6.45% was obtained. The SG from RRM were slightly higher than CSM, which, in some scenarios and environments, has a higher predict gain but is overall below the MTM and RRM predicted gains (Fig S1.5).

Table 3. Predicted selection gains (in percentage) by the three models: compound symmetry (CSM), multiple-trait (MTM) and random regression (RRM).

Selection intensity	E1	E2	E3	E4
		CSM		
5	21.79	20.07	13.11	13.49
10	15.65	14.48	8.95	10.76
15	13.17	11.02	7.82	8.92
20	11.43	9.33	6.45	6.98

MTM				
5	25.54	20.29	13.91	15.69
10	19.29	15.35	10.78	12.26
15	16.05	12.38	8.92	10.50
20	13.75	10.56	7.74	9.12
RRM				
5	25.06	20.29	13.00	14.74
10	18.86	15.35	9.78	11.45
15	14.86	12.29	8.43	10.27
20	12.95	10.48	7.16	8.90

Discussion

~~Akaike information criterion (AIC) is among the most adopted statistical criterion for model selection, ranking models based on efficiency [31]. In addition, in RRM, when selecting the polynomial order, AIC has been widely used [31]. Moreover, many studies adopted AIC as criterion for model selection [32–34]. Based on AIC, the~~ RRM was superior over MTM and CSM, being ~~a~~ best fitted model ~~in our study~~. ~~The LRT is based on the difference between the maximum likelihood under the null and under the alternative hypothesis (i.e. when the referred effect is not considered).~~ Our results indicated that the genotypic and GEI effects were significant, by LRT.

The MTM and RRM ~~used in this study~~ allows modeling the genetic and residual covariance structures, ~~aiming to better understand these effects~~. The adequate modeling of genotypic and residual effects is a way to mitigate the GEI effects and maximize the selective accuracy [35–37]. Studies with maize, potato, and wheat achieve more than 5% of gain in the selective accuracy when modelled the GEI effect [38]. The heterogeneous

residual variance structure was the best fitted in all models (*CS.Rhe*, *MT.Rhe*, and *RR.2.Rhe*), being models with this residual structure stated by some studies in annual crops [33,39,40]. In addition, the RRM best fitted was *RR.2.Rhe* which is the Legendre polynomials of second order for the genetic effect.

~~After selection of the best fitted model for the three methods studied, the estimated genetic and non-genetic parameters were compared.~~ The estimated residual variance presented greater disparity, in all environments. As it presented by MTM, where the environment E3 presented values 60% higher than those from the environment E4 ~~(Table 2)~~. Similar results were obtained by CSM and RRM ~~(Table 2).~~ This discrepancy reinforces the results found by AIC and supports the choice for the models with heterogeneous residual variance.

The genotypic correlation among environments, given by CSM, below 0.75 demonstrate the necessity of more robust analysis for the genetic selection [7]. Moreover, the CSM assumes a constant genetic variances and correlations between environments equal to one, what are not a prerequisite in MTM and RRM [10]. In this way, when complex GEI occurs, models that incorporate precise variance structures should be preferred, such as MTM and RRM [7], which demonstrates the fragility of CSM.

The evaluation of GEI can be developed through several methods [14]. Alves et al. [6] believe that new methodologies based on mixed models achieve most effectivity in the genetic selection, such as Factor Analytic Multiplicative Mixed Models [41] and RRM. The most complex and parameterized model, i.e. MTM, estimates each random effect for each environment, simultaneously. Additionally, covariances between each pair of environments are also estimated. RRM also estimates individual variance components for each environment and covariance between each pair of environments. It has been applied to capture the change of a trait continually over growth stages (in perennial

species) or environmental gradients (in MET), with fewer parameters to be estimated, compared to MTM [8,11]. In our study, the MTM and RRM estimated 14 and 7 parameters, respectively, whereas the CSM presented 6 parameters. The parsimony (i.e. lower number of parameters) is the only advantage of CSM over MTM and RRM.

The CSM allowed estimate the GEI variance and, its division by the phenotypic variance is the coefficient of determination of GEI effects. In this study, CSM, indicated complex GEI effect (i.e. significant GEI effects by LRT), as well as in RRM (i.e. polynomial order 2). In the random regression context, the GEI effects can be detected through reaction norms [30]. The GEI occurs when the reaction norms intersect, diverge, or converge [30]. Our study verified intersections of reaction norms through environmental gradient for many genotypes. Also, it can be noted in some relevant studies with elephant-grass and eucalyptus, indicates the presence of complex GEI in the reaction norms [6,42]. In the MTM, the GEI effects cannot be direct estimated, since these effects are confounded with the genetic effects ($\hat{\sigma}^2_{g_i} = \hat{\sigma}^2_{g_i} + \hat{\sigma}^2_{ge_i}$). However, if the genotype ranking differs among environments, the complex GEI occurs. Volpato et al. [43] demonstrate the presence of this effect in genotype ranking, by MTM.

As MTM presents an unstructured matrix for the random effects, it is able to estimate variance components per environment and covariance between pair of environments, being considered a complete model. Although the algorithms AI (average information) or EM (expectation-maximization) are widely applied in the estimation process, they face some issues regarding convergence for unstructured matrix, in MTM [8]. Conversely, the estimation process of random effects, in RRM, requests a reduced structure and fewer parameters, comparing to MTM [6,8,11]. Our study presents satisfactory results for both models, however, in case of larger number of environments,

MTM would become prohibitive. In addition, RRM has the capability to estimate continuous variance function through the observed genotypic effects matrix.

The heritability values are more accurate in MTM and RRM than in CSM, because these models consider heterogeneity of genetic variances across environments [6]. This characteristic carries out to more accurate, and higher values of heritabilities were estimated by MTM and RRM in this study. Regarding heritability estimation, the superiority of RRM over CSM is also reported by Alves et al. [6]. This parameter can be affected by any change in the variance components, i.e. genetic or residual variances [44]. In addition, this parameter is of interest in any breeding program, since the genotypes selection will consider their predicted values, based on heritabilities estimates [45].

In genetic evaluation context, the selective accuracy is the most important statistical parameter, as mentioned by Henderson [46]. It is able to provide the right ranking of genotypes [46]. Also, this parameter is defined as the correlation between predicted and real genotypic values [47]. In MTM, the increase of accuracy is proportional to the difference between genetic and environmental correlations of the trait [48], and one of the advantages of MTM is providing the higher selective accuracy [8,49]. Resende and Duarte [15] state that accuracy is a way to report about the experimental quality, since this parameter consider residual and genetic variances. According to Resende and Duarte [15], in MTM, a very high selective accuracy was estimated for the environment E2 ~~(Table 2).~~ On the other hand, in RRM, the environment E4 presented high selective accuracy, while the other environments presented very high accuracies ~~(Table 2).~~

Regarding to selection gain, and considering the above-mentioned parameters, MTM and RRM presented slightly superiority in gains, as they estimated one genotypic value per environment and considers the genotypic correlation between pair of

environments. These methods reach 25% of gain when selecting 5 genotypes ~~(Table 3).~~ The environments E1 and E2 stood out for gains, which were over 5% for other environments, when comparing the same selection intensity ~~(Table 3).~~ Conversely, for CSM, the worst environment (E3) revealed low gains from 6.45% to 13.11%, when selected 5 to 20 genotypes, respectively. This difference among models can be explained by the variance component estimates, which infer a higher heritability (h^2), for MTM and RRM, based on the characteristic of the environment (biotic and abiotic stress) and experimental quality (high selective accuracy and experimental coefficient of variation) [5,15]. It is important to highlight that the selective accuracies of CSM, which were, in some cases, even higher than MTM and RRM. This can be interpreted as a negative aspect of CSM, which does not consider the difference between genotypic variance ($\hat{\sigma}_g^2$) in each environment, since it considers only the overall genotypic variance and GEI variance, providing misestimated accuracies. For instance, the lower experimental quality for environment E4, compared to the others (E1, E2 and E3) in this study, is confirmed by experimental coefficient of variation and selective accuracy for MTM and RRM ~~(Table 2)~~, which is not detected in CSM estimates.

The difference among ~~the~~ genotypes ranking, in all models, is due the GEI effect, which affects the performance of the genotypes in different environments [14]. As mentioned by van Eeuwijk et al. [30], when genes express differently over environments, the GEI will occur. So, the different rankings ~~(Table S1.4)~~ are another proof of GEI effect in our study, for all models. Finally, the presence of some inter-population hybrids is observed in the ranking, figuring out among the top commercial hybrids, which reveals the high potential of these genotypes ~~(Table S1.4~~). The breeder can focus on the parental hybrids of these genetic materials, and, in which environments they performed better, to recommend basing on each one environment individually.

The best models to adopt are ~~the~~ MTM and RRM, as they allowed to estimate the genotypic variance ($\hat{\sigma}_g^2$) per environment. Nevertheless, the heterogeneous structure of CSM provides residual variance ($\hat{\sigma}_e^2$) per environments. But, the absence of genetic variance estimates per environment prevents CSM from providing genetic correlation among environments, which can be a disadvantage comparing to MTM and RRM. Even so, all these methods provide estimates of variance components and genetic parameters per environment. For maize breeding, GEI is a relevant effect and the development of genetic materials must consider it [4,5].

Conclusion

The RRM was superior over MTM and CSM by AIC for dealing with MET, being a best fitted model. Although MTM and RRM present detailed variance components and genetic parameter estimates, based on the selective accuracies and heritabilities estimates, the RRM was slightly superior. These models have the capability to mitigate all random effects of the model. RRM also offers reliable results and can reduce the number of parameters, besides the convergence issues. In this case, to solve a potential overflow issue of estimates, in MTM, the RRM is superior. In practical terms, the MTM presented slightly higher gains, what can be justified by the computational efforts required by the model. It becomes prohibitive when there is higher number of evaluated environments. Finally, interpopulational hybrids present better performances over some commercial hybrids, in some environments.

References

1. USDA. Economic Research Service. 2019.

2. Resende MDV. Genética quantitativa e de populações. Suprema, Visconde do Rio Branco. 1st ed. 2015; 463.

3. Marchal A, Schlichting CD, Gobin R, Balandier P, Millier F, Muñoz F, et al. Genotype by environment interactions in forest tree breeding: review of methodology and perspectives on research and application. Plant Genome. 2019;13: 281–288. doi:10.1017/S0021859605005587

4. Bernardo R. Breeding for Quantitative Traits in Plants. 2nd ed. Woodbury, Minnesota: Stemma Press; 2010.

5. Lima RO de, Borém A. Melhoramento de Milho. Viçosa - MG: Editora UFV; 2018.

6. Alves RS, de Resende MDV, Azevedo CF, Silva FF e, Rocha JR do AS de C, Nunes ACP, et al. Optimization of Eucalyptus breeding through random regression models allowing for reaction norms in response to environmental gradients. Tree Genet Genomes. 2020;16: 38. doi:10.1007/s11295-020-01431-5

7. Resende MDV de. Matemática e estatística na análise de experimentos e no melhoramento genético. Embrapa Florestas, Colombo; 2007.

8. Resende MD V, Silva FF, Azevedo CF. Estatística matemática, biométrica e computacional: modelos mistos, multivariados, categóricos e generalizados (REML/BLUP), inferência bayesiana, regressão aleatória, seleção genômica, QTL-QWAS, estatística espacial e temporal, competição, sobrevivência. Suprema, Visconde do Rio Branco. 2014;1: 881.

9. Sun J, Rutkoski JE, Poland JA, Crossa J, Jannink JL, Sorrells ME. Multitrait, random regression, or simple repeatability model in high-throughput phenotyping data improve genomic prediction for wheat grain yield. Plant Genome. 2017;10. doi:10.3835/plantgenome2016.11.0111

10. Meyer K. Random regressions to model phenotypic variation in monthly weights of Australian beef cows. Livest Prod Sci. 2000;65: 19–38. doi:10.1016/S0301-6226(99)00183-9

11. Rocha JR do AS de C, Marçal T de S, Salvador FV, da Silva AC, Machado JC, Carneiro PCS. Genetic insights into elephantgrass persistence for bioenergy purpose. PLoS One. 2018;13: 1–16. doi:10.1371/journal.pone.0203818

12. Kirkpatrick M, Heckman N. A quantitative genetic model for growth, shape, reaction norms, and other infinite-dimensional characters. J Math Biol. 1989;27: 429–450. doi:10.1007/BF00290638

13. Meyer K. Modeling "repeated" records : Covariance functions and random regression models to analyse animal breeding data. Proceedings of the 6th World Congress on Genetics Applied to Livestock Production. 1998. pp. 517–520.

14. Li Y, Suontama M, Burdon RD, Dungey HS. Genotype by environment interactions in forest tree breeding: review of methodology and perspectives on research and application. Tree Genet Genomes. 2017;13. doi:10.1007/s11295-017-1144-x

15. Resende MDV De, Duarte JB. Precisão e controle de qualidade em experimentos de avaliação de cultivares. Pesqui Agropecuária Trop. 2007;37: 182–194. doi:10.5216/pat.v37i3.1867

16. Martin JGA, Nussey DH, Wilson AJ, Réale D. Measuring individual differences in reaction norms in field and experimental studies: a power analysis of random regression models. Methods Ecol Evol. 2011;2: 362–374. doi:10.1111/j.2041-210X.2010.00084.x

17. Brommer JE, Kontiainen P, Pietiäinen H. Selection on plasticity of seasonal life-history traits using random regression mixed model analysis. Ecol Evol. 2012;2:

695–704. doi:10.1002/ece3.60

18. Mota RR, Tempelman RJ, Lopes PS, Aguilar I, Silva FF, Cardoso FF. Genotype by environment interaction for tick resistance of Hereford and Braford beef cattle using reaction norm models. Genet Sel Evol. 2016;48: 3. doi:10.1186/s12711-015-0178-5

19. Arnold PA, Kruuk LEB, Nicotra AB. How to analyse plant phenotypic plasticity in response to a changing climate. New Phytol. 2019;222: 1235–1241. doi:10.1111/nph.15656

20. Alvares CA, Stape JL, Sentelhas PC, de Moraes Gonçalves JL, Sparovek G. Köppen's climate classification map for Brazil. Meteorol Zeitschrift. 2013;22: 711–728. doi:10.1127/0941-2948/2013/0507

21. Cruz JC, Karam D, Monteiro MAR, Magalhães PC. A cultura do milho. Sete Lagoas, MG: Embrapa Milho e Sorgo; 2008.

22. Gilmour AR, Gogel BJ, Cullis BR, Welham Sj, Thompson R. ASReml user guide release 4.1 structural specification. Hemel hempstead VSN Int ltd. 2015.

23. Schaeffer LR. Random Regression Models. 2016; 171.

24. Akaike H. A new look at the statistical model identification. Selected Papers of Hirotugu Akaike. Springer; 1974. pp. 215–222.

25. Wilks SS. The Large-Sample Distribution of the Likelihood Ratio for Testing Composite Hypotheses. Ann Math Stat. 1938;9: 60–62. Available: https://www.jstor.org/stable/pdf/2957648.pdf

26. Kirkpatrick M, Lofsvold D, Bulmer M. Analysis of the inheritance, selection and evolution of growth trajectories. Genetics. 1990;124: 979–993.

27. Resende MDV. Software Selegen-REML/BLUP: a useful tool for plant breeding. Crop Breed Appl Biotechnol. 2016;16: 330–339. doi:10.1590/1984

28. R Core Team. R: A language and environment for statistical computing. Vienna, Austria: R Foundation for Statistical Computing; 2019.

29. Butler DG, Cullis BR, Gilmour AR, Gogel BJ. ASReml-R reference manual (version 3). The State of Queensland, Department of Primary Industries and Fisheries: Brisbane, Qld. 2009.

30. Van Eeuwijk FA, Bustos-Korts D V., Malosetti M. What should students in plant breeding know about the statistical aspects of genotype × Environment interactions? Crop Sci. 2016;56: 2119–2140. doi:10.2135/cropsci2015.06.0375

31. Corrales JD, Munilla S, Cantet RJC. Polynomial order selection in random regression models via penalizing adaptively the likelihood. J Anim Breed Genet. 2015;132: 281–288. doi:10.1111/jbg.12130

32. Faveri J De, Verbyla AP, Pitchford WS, Venkatanagappa S, Cullis BR. Statistical methods for analysis of multi-harvest data from perennial pasture variety selection trials. Crop Pasture Sci. 2015;66: 947. doi:10.1071/CP14312

33. Melo VL de, Marçal T de S, Rocha JRAS de C, dos Anjos RSR, Carneiro PCS, Carneiro JE de S. Modeling (co)variance structures for genetic and non-genetic effects in the selection of common bean progenies. Euphytica. 2020;216: 77. doi:10.1007/s10681-020-02607-9

34. Abrantes KKB, Paiva LM, Almeida RG de, Urbano E, Ferreira AD, Mazucheli J. Modeling the individual height and volume of two integrated crop-livestock-forest systems of *Eucalyptus* spp. in the Brazilian Savannah. Acta Sci Agron. 2019;41: 42626. doi:10.4025/actasciagron.v41i1.42626

35. Smith A, Cullis B, Thompson R. Analyzing Variety by Environment Data Using Multiplicative Mixed Models and Adjustments for Spatial Field Trend. Biometrics. 2001;57: 1138–1147. doi:10.1111/j.0006-341X.2001.01138.x

36. Smith AB, Cullis BR, Thompson R. The analysis of crop cultivar breeding and evaluation trials: An overview of current mixed model approaches. J Agric Sci. 2005;143: 449–462. doi:10.1017/S0021859605005587

37. Smith AB, Ganesalingam A, Kuchel H, Cullis BR. Factor analytic mixed models for the provision of grower information from national crop variety testing programs. Theor Appl Genet. 2015;128: 55–72. doi:10.1007/s00122-014-2412-x

38. Burgueño J, Crossa J, Cotes JM, Vicente FS, Das B. Prediction Assessment of Linear Mixed Models for Multienvironment Trials. Crop Sci. 2011;51: 944–954. doi:10.2135/cropsci2010.07.0403

39. Rocha JR do AS de C, Nunes KV, Carneiro ALN, Marçal TDS, Salvador FV, Carneiro PCS, et al. Selection of Superior Inbred Progenies toward the Common Bean Ideotype. Agron J. 2019;1181–1189: 1181–1189. doi:10.2134/agronj2018.12.0761

40. So YS, Edwards J. Predictive ability assessment of linear mixed models in multienvironment trials in corn. Crop Sci. 2011;51: 542–552. doi:10.2135/cropsci2010.06.0338

41. Resende MDV de, Thompson R. Factor analytic multiplicative mixed models in the analysis of multiple experiments. Rev Matemática e Estatística. 2004;22: 31–52.

42. Rocha VD, Tiago PV, Tiago AV, Pedri ECM, Cardoso ES, Rossi AAB. Genetic diversity among Hymenaea courbaril L. genotypes naturally occurring in the north of Mato Grosso State, Brazil. Genet Mol Res. 2017;16. doi:10.4238/gmr16039706

43. Volpato L, Alves RS, Teodoro PE, De Resende MDV, Nascimento M, Nascimento ACC, et al. Multi-trait multi-environment models in the genetic

selection of segregating soybean progeny. PLoS One. 2019;14: 1–22. doi:10.1371/journal.pone.0215315

44. Batista CM, Freitas MLM, de Moraes MA, Zanatto ACS. Estimates of genetic parameters and variability in provenances and progeny of Handroanthus velossoi. Pesqui Florest Bras. 2012;32: 269–276. doi:0.4336/2012.pfb.32.71.269

45. Maia MCC, Resende MDV de, Paiva JR de, Cavalcanti JJV, Barros L de M. Seleção simultânea para produção , adaptabilidade e estabilidade genotípicas em clones de cajueiro , via modelos mistos 1. Pesqui Agropecuária Trop. 2009;39: 43–50.

46. Henderson CR. Applications of linear models in animal breeding. University of Guelph Guelph; 1984.

47. Van Vleck LD, Pollak EJ, Oltenacu EAB. Genetics for the Animal Sciences. New York: W.H. Freeman and Company; 1987.

48. Thompson R, Meyer K. A review of theoretical aspects in the estimation of breeding values for multi-trait selection. Livest Prod Sci. 1986;15: 299–313. doi:10.1016/0301-6226(86)90071-0

49. Okeke UG, Akdemir D, Rabbi I, Kulakow P, Jannink JL. Accuracies of univariate and multivariate genomic prediction models in African cassava. Genet Sel Evol. 2017;49: 1–10. doi:10.1186/s12711-017-0361-y

Supporting information

Table S1.1. Location of environments, with the corresponding geographic coordinates and altitudes.

Table S1.2. Complete diallel scheme involving thirteen F_2 population (used as parents) and their respectively crosses (H).

Table S1.3. Selective accuracy by each genotype, per environment, for: Compound Symmetry (CSM, Multiple-Trait (MTM) and Random Regression (RRM) models.

Table S1.4. Ranking for compound symmetry (STM), multiple-trait (MTM) and random regression (MTM) models, in each environment (E1 to E4). Until the dashed line are the top ten hybrids, and it is highlighted the inter-population hybrids into it.

Fig S1.5. Coincidence index, for all environments in all methodologies. The compound symmetry (CSM), multiple-trait (MTM) and random regression (MTM) models, are followed by each environment (_1, _2, _3 and _4). The letters A, B, C and D correspond to each scenario (5, 10, 15, and 20, respectively).

Figure 2

Click here to access/download;Figure;Figure_02(TIF).tif

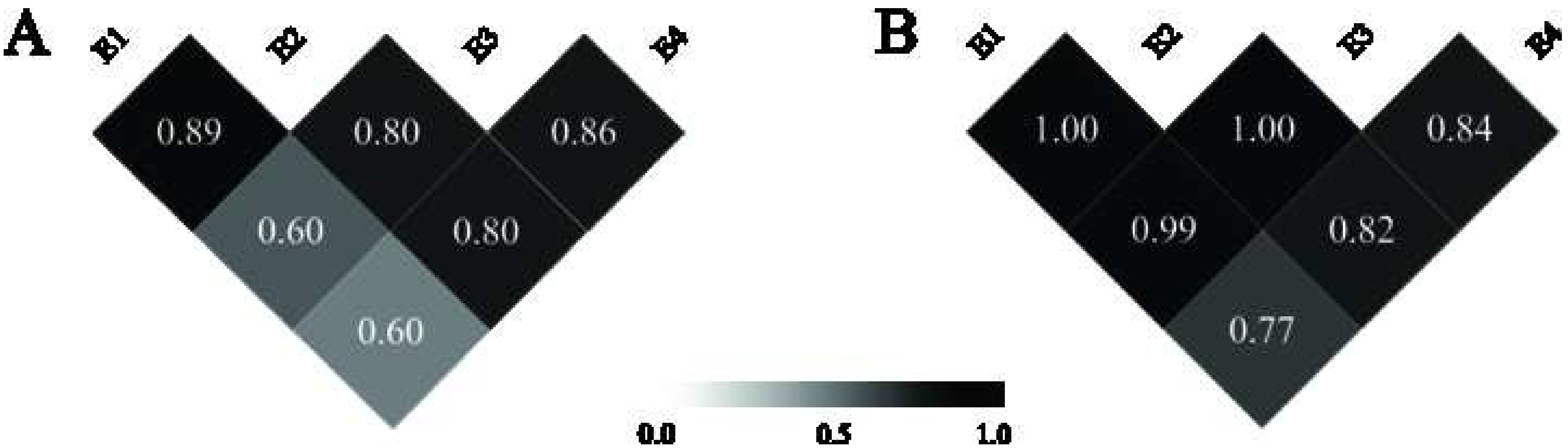

Click here to access/download;Figure;Figure_03(tif).tif

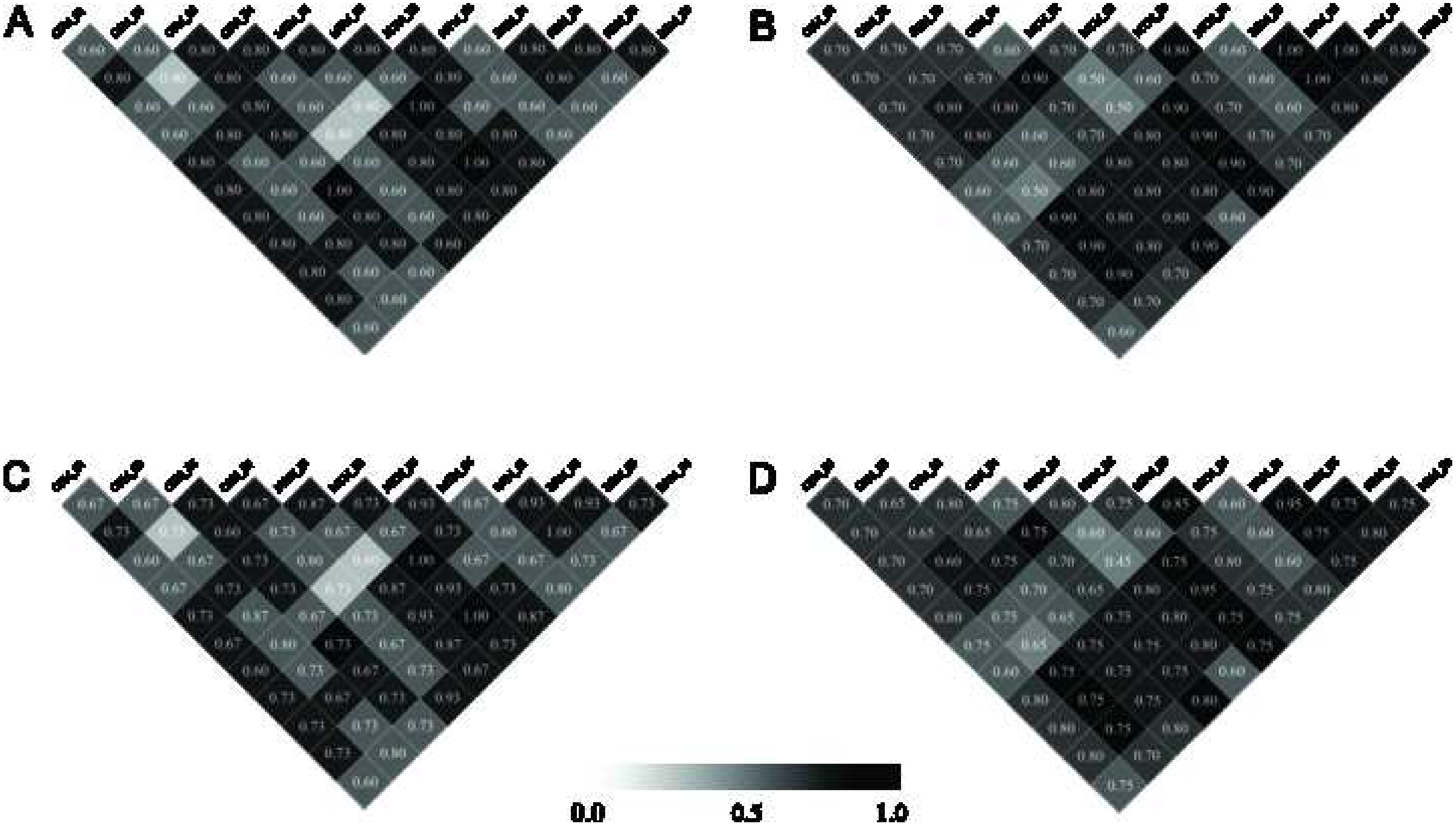

Figure 1

Click here to access/download;Figure;fig1300.tif

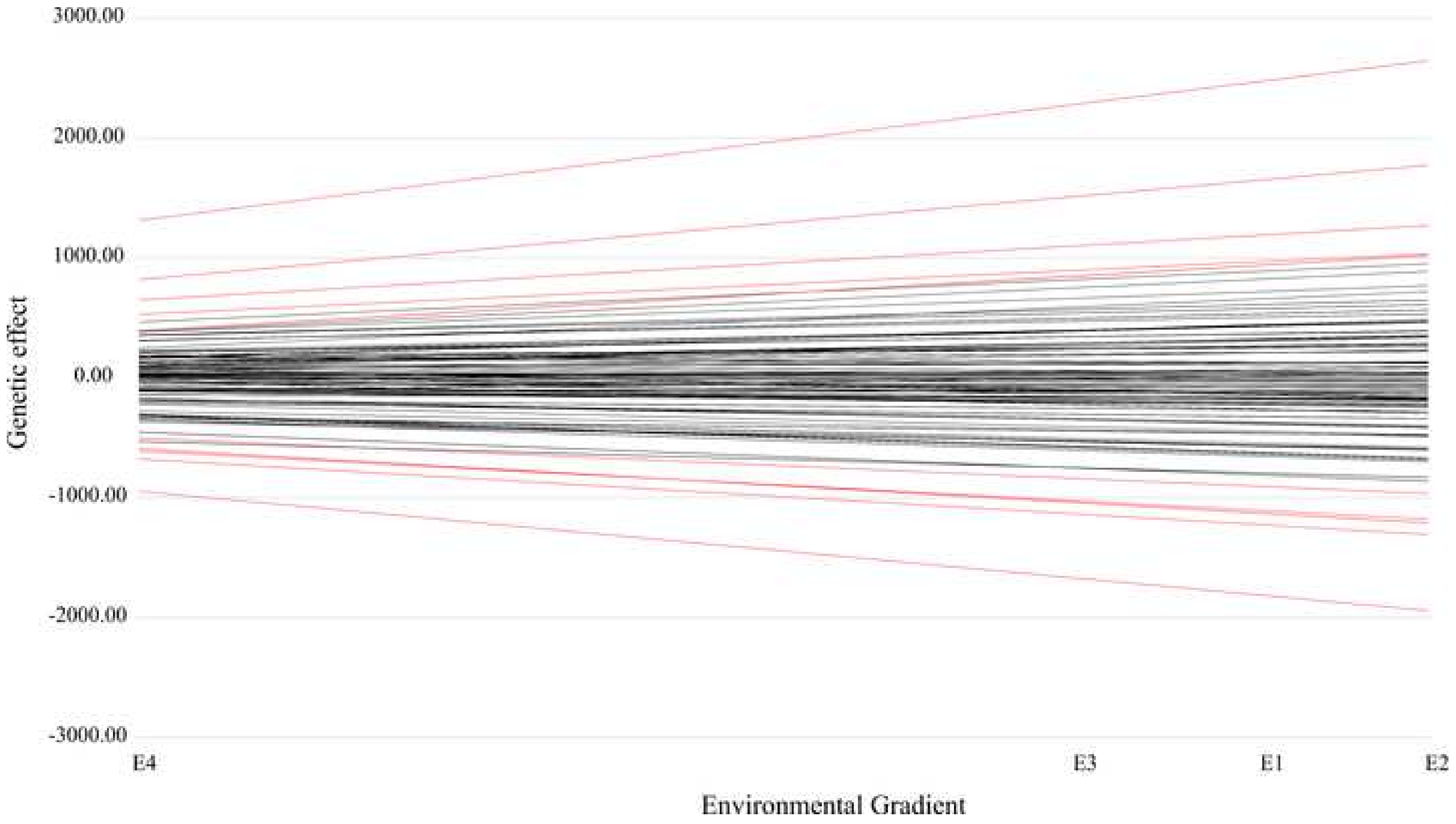

Dataset

Click here to access/download
Supporting Information
Dataset_IFC.txt

Supplementary material

Click here to access/download
Supporting Information
Supporting Information.docx

Multiple-trait, random regression, and compound symmetry models for analysis of multi-environment trials in maize breeding

Igor Ferreira Coelho[1] | Marco Antônio Peixoto[1] | Jeniffer Santana Pinto Coelho Evangelista[1] | Suellen Sales[1] | Rodrigo Silva Alves[1] | Jefferson Fernando Naves Pinto[2] | Edésio Fialho dos Reis[2] | Marcos Deon Vilela de Resende[3] | Leonardo Lopes Bhering[1*]

[1] Universidade Federal de Viçosa (UFV), Departamento de Biologia Geral, Viçosa, CEP: 36570-900, Minas Gerais, Brazil

[2] Universidade Federal de Jataí (UFJ), Departamento de Agronomia, Jataí, CEP: 75801-615, Goiás, Brazil

[3] Embrapa Café / Universidade Federal de Viçosa (UFV), Departamento de Estatística, Viçosa, CEP: 36570-900, Minas Gerais, Brazil

[*] Corresponding author: leonardo.bhering@ufv.br – +55 31 3612-5003

Keywords

REML; BLUP; Mixed models; covariance structures; genotype-by-environment interaction; genetic selection; selective accuracy.

Abstract

Modelling covariance structures is a good way to improve statistical capability to make more accurate estimations and predictions in multi-environmental trials (MET) analysis. By modelling the random terms of the statistical model, as genetic, genotype-by-environment interaction (GEI) and residual effects, there will be more accurate predictions, and the reliability in the genetic selection tends to increase. In this way, this study compared the multiple-trait (MTM), random regression (RRM), and compound symmetry (CSM) models, and their homogeneous and heterogeneous residual structures. For this, phenotypic data of 84 hybrids of maize tested in four environments were used. The goodness-of-fit were indicated for each model by Akaike information criterion (AIC). Then, we evaluated the variance components, genetic parameters, and genetic gains considering four intensities of selection (5, 10, 15 and 20 genotypes), over the four environments assessed. According to AIC, the CSM, RRM (Legendre Polynomial of order 2) and MTM with heterogeneous residual variance presented the best fits. CSM presented a unique genotypic variance and GEI variance estimate, having just the residual variances estimated per environment. Conversely, MTM and RRM were capable to estimate a genotypic variance for each environment. This characteristic carried out the MTM and RRM to higher heritability values (>0.40), while the heritabilities estimated by CSM were lower (<0.27). Similar performance was observed in selective accuracy values where CSM presented inferior values when compared with MTM and RRM for all environments, excepted for one out of four environments, what could be a super-estimated value by CSM. The selection gains followed the same pattern than the previous parameters, where CSM presented the lowest predicted gains and MTM presented the highest predicted gains, since it is the more complete model. RRM was close to MTM

selection gains, achieving gains around 25%. Thus, the RRM presented better results, due to the parsimonious, and, was the best suited model to deal MET data in maize breeding.

Introduction

Maize (*Zea mays* L.) is the most cultivated crop worldwide, with world yield of 1.1 billion of tons in the 2018/2019 crop year [1]. An important advantage of this crop is the capacity of being cultivated in a range of environments and different seasons. Such aspects lead to differential response by genotypes to different environments, which is known as genotype-by-environment interaction (GEI) [2], or even as phenotypic plasticity [3].

Due to environmental effects on quantitative traits, performance tests of potential genotypes are conducted in multiple years and locations. The variation in performance depends on the magnitude of GEI, which occurs when there is variation in the genotype relative performance of the genotype across the environments tested [4]. An "environment" can be defined by biotic or abiotic aspects to which the plants are exposed. There are also other characteristics used to define environments, such as technology level or plant population density [5].

The compound symmetry model (CSM) is the basic model, while the multiple-trait model (MTM) is the most complete to deal with GEI. Although the CSM is a simple and parsimonious structure, having a small number of parameters to be estimated this model have limited assumptions, such as correlation equal to 1 and unique genetic variance [6]. Moreover, MTM (unstructured model) tends to present problems regarding convergence, due to the high number of parameters to be estimated [7–9]. Then, the MTM became prohibitive when the number of environments is high [8].

In this sense, the performance of the random regression model (RRM) is similar to the MTM, whereas estimating less parameters [10]. RRM captures the continuous changing of a trait, over time or environmental gradient, using fewer parameters than MTM [9,11]. It is a classical quantitative model extension, which predicts the genetic (additive, dominance, and epistasis) component of reaction norms. The prediction of genotypic values can be also made for untested environments which fit into the environmental gradient [6]. RRM is suitable to fit complex reaction norms [12,13]. The reaction norms considers the GEI and can identify its underlying causes, being plotted over the environmental gradient (X axis) [14]. Next, these reaction norms fitted using RRM assign the predicted genotypic effect by using the random regression coefficients to build a function per genotype, according to the environmental gradient [8]. Then, there will be a genotypic value for each environment, considering GEI and the genetic covariances and residual variance changes over the environmental gradient.

Selective accuracy is the most suitable alternative to compare different statistical methods in the genetic improvement [15]. Moreover, selection gain and heritability are also used to compare methodologies which deal with GEI, by evaluating the variance components and genotypic values [5]. Maximizing the selection gain is an important goal of any breeding program. The selection gain predicts the performance that these genetic materials will provide for the trait in the following cycle (with recombination) of breeding. Accuracy gives the certainty of choosing the right progenies. Besides accuracy, it is necessary to understand the genetic basis, selection units, and heritability of a trait. It is applied for some breeding strategies, such as recurrent selection methods, for decision making in breeding programs [5].

Considering the expansion of maize crop cultivation, studies on GEI are necessary to evaluate different models dealing with this effect and indicate the most suitable to

handle with MET data to maximize the selective accuracy and the gains with selection [9]. Additionally, RRM studies have been developed to evaluate plasticity and GEI in animals and plant science, providing satisfactory results [6,16–19]. Thus, this study aims: (i) evaluate and compare three models for MET analysis: CSM, MTM, and RRM; (ii) determine which model is the best suited to estimate variance components and selective accuracies, and predict genotypic values and selection gains.

Material and Methods

Experimental data

The trials were carried out between January and July 2018, in four sites, in Goiás, Brazil (Table S1.1). The environments were considered as each evaluated site, totaling four environments (hereafter E1, E2, E3, and E4). The region presents wet temperate, with dry winters and hot summers (Cwa) [20]. Average annual temperature is around 21.5°C and average rainfall is between 1.400 and 2.000 mm year^{-1}. The agricultural practices were based on those used for maize cultivation in Brazil [21].

A data set with 78 inter-population hybrids (Table S1.2) and the six commercial hybrids (F_1) most used in the region was used. The inter-population hybrids were developed from 13 F_2 populations, derived from commercial hybrids widely adopted in the region. The trials were conducted in complete block design with three replications and 44 plants per plot. Plots consisted of four 4 m rows, with a spacing of 0.40 m between plants and 0.45 m between rows. To eliminate the competition effect, only the two central rows were evaluated. The grain yield (GY) trait was evaluated by the weight of the plot and converted to hectares (kg ha^{-1}).

Statistical analyses

Three statistical models were fitted: (i) Compound symmetry model (CSM), (ii) Multiple-trait model (MTM), and (iii) Random regression models (RRM) fitted through Legendre polynomials.

The estimation of variance components and prediction of the genotypic values for GY trait were made via restricted maximum likelihood/best linear unbiased prediction (REML/BLUP) procedure [22].

i - Compound symmetry model

The CSM was given by the following equation:

$$\mathrm{y} = \mathbf{X}r + \mathbf{Z}g + \mathbf{W}ge + e,$$

where *y* is the vector of phenotypes; *r* is the vector of block-environment combinations (assumed to be fixed), which comprises the effects of environment and block within environment, added to the overall mean; *g* is the vector genotypic effects (assumed as random), *ge* is the vector GEI effects (random), and *e* is the vector of residuals (random). Uppercase bold letters refer to the incidence matrices for those effects.

In this model: $g \sim N(0, I\sigma_g^2)$, $ge \sim N(0, I\sigma_{ge}^2)$, and $e \sim N(0, R)$; where σ_g^2 is the genotypic variance between genotypes, σ_{ge}^2 is the GEI variance, I is an identity matrix with appropriate order to the respective effect, and R refers to a diagonal matrix of residual variances. Models accounting for different residual variance structures (homogeneous and heterogeneous) were tested here.

ii - Multiple-trait model

The MTM was given by the following equation:

$$\mathrm{y} = \mathbf{X}r + \mathbf{Z}g + e.$$

In this model: $g \sim N(0, \Sigma_g \otimes I)$, and $e \sim N(0, R)$; where Σ_g is the genotypic covariance matrix, I is an identity matrix with appropriate order, $\otimes$ denotes the Kronecker product, and R refers to a diagonal matrix of residual variances. Models accounting for different residual variance structures (homogeneous and heterogeneous) were tested here.

iii - Random regression models

In order to use Legendre polynomials, the phenotypic mean of each environment (μ_i) must be scaled to range from -1 to +1. The environmental gradient values (E_i) were obtained by the following expression [23]:

$$E_i = -1 + 2[(\mu_i - \mu_{min})/(\mu_{max} - \mu_{min})].$$

The RRM were fitted through Legendre polynomials, considering all possible degrees of fit, as follows:

$$Y_{ijk} = \mu + S_j + R(S_{jk}) + \sum_{d=0}^{D} \alpha_{id} \Phi_{ijd} + e_{ijk},$$

where Y_{ijk} is the ith genotype (i = 1, 2, …, 84) in the jth environment (j = 1, 2, 3, 4) in the kth block (k = 1, 2, 3); μ is the overall mean; S_j is the fixed effect of environment j; $R(S_{jk})$ is the fixed effect of block k within environment j; d is the polynomial degree, ranging from *0* to *D* (*D = number of environments - 1*); α_{id} is the random regression coefficient for the orthogonal polynomial for the genotype effects; Φ_{ijd} is the dth orthogonal polynomial for the jth environment for the ith genotype; and e_{ijk} is the residual random effect associated with Y_{ijk}.

In the matrix notation, the above model was described by the following equation:

$$y = \boldsymbol{X}r + \boldsymbol{Z}g + e.$$

In this model: $g \sim N(0, K_g \otimes I_{84})$, and $e \sim N(0, R)$; where K_g is the covariance matrix for the coefficients of genotypic effects, $\otimes$ denotes the Kronecker product, I_{84} is an identity matrix of order *84*, and R refers to a diagonal matrix of residual variances.

Models accounting for different residual variance structures (homogeneous and heterogeneous) were tested here.

Model selection

In order to compare the residual variance structures in CSM, MTM, and RRM the Akaike information criterion (AIC) [24] was used. Significance of the random effects of the three models (CSM, MTM, and RRM) were tested by the likelihood ratio test (LRT) for [25].

Variance components and genetic parameters

For CSM, phenotypic variance ($\hat{\sigma}_p^2$), broad-sense heritability (h^2), coefficient of determination of GEI (c_{ge}^2), and genotypic correlation among environments (r_{gloc}), were estimated using the following equations:

$$\hat{\sigma}_p^2 = \hat{\sigma}_g^2 + \hat{\sigma}_{ge}^2 + \hat{\sigma}_e^2,$$

$$h^2 = \frac{\hat{\sigma}_g^2}{\hat{\sigma}_p^2},$$

$$c_{ge}^2 = \frac{\hat{\sigma}_{ge}^2}{\hat{\sigma}_p^2}, \text{ and}$$

$$r_{gloc} = \frac{\hat{\sigma}_g^2}{\hat{\sigma}_g^2 + \hat{\sigma}_{ge}^2}.$$

For RRM, the estimates of the genotypic variance ($\hat{\sigma}_g^2$) and predicted genotypic values ($\tilde{g}_{ij}$), in the original scale, were estimated/predicted by the following expressions [23,26]:

$$\hat{\sigma}_g^2 = \Phi_{ijd}\hat{K}_g\Phi'_{ijd}, \text{ and}$$

$$\tilde{g}_{ij} = \sum_{d=0}^{D}\hat{\alpha}_{id}\Phi'_{ijd}.$$

For MTM and RRM, phenotypic variance ($\hat{\sigma}_p^2$) and broad-sense individual heritability (h^2) were estimated by the following expressions [8]:

$$\hat{\sigma}_p^2 = \hat{\sigma}_g^2 + \hat{\sigma}_e^2, \text{ and}$$

$$h^2 = \frac{\hat{\sigma}_g^2}{\hat{\sigma}_p^2}.$$

The selective accuracy ($r_{\hat{g}g}$) was calculated, for CSM, MTM and RRM, by the following expressions, respectively:

$$r_{\hat{g}g} = \sqrt{1 - \frac{\left[1-\left(1-\frac{PEV}{\hat{\sigma}_g^2}\right)\right]\left[1-\left(1-\frac{PEV}{\hat{\sigma}_{ge}^2}\right)\right]}{1-(1-\frac{PEV}{\hat{\sigma}_g^2})(1-\frac{PEV}{\hat{\sigma}_{ge}^2})}},$$

$$r_{\hat{g}g} = \sqrt{1 - \frac{PEV}{\hat{\sigma}_g^2}}, \text{ and}$$

$$r_{\hat{g}g} = \sqrt{1 - \frac{\Phi_{ijd} PEV \Phi'_{ijd}}{\hat{\sigma}_g^2}},$$

where PEV is the prediction error variance, obtained by the diagonal elements of the inverse of the coefficient matrix (information matrix) of the mixed model equations.

The experimental coefficient of variation (CV_e) was calculated using the $\hat{\sigma}_e$ of each environment:

$$CV_e = \frac{\hat{\sigma}_e}{\mu},$$

where μ is the phenotypic mean of the experiment.

Selection gain

Rankings per environment were carried out for each method. Then, selection gain (*SG*) was predicted, for each environment, considering the selection of 20, 15, 10 and 5 genotypes, based on the following expression [27]:

$$SG\ (\%) = \left(\frac{[(\Sigma_{i=1}^{n} GV_i)/n] - \mu_p}{\mu_p}\right) x\ 100,$$

where: GV_i is the predicted genetic value of genotype i, n is the number of genotypes selected, and μ_p is the overall mean of the population.

Software

All analyses were conducted in the ASReml 4.1 [22] and ASReml-R, implemented in the R software [28,29].

Results

Model selection and significance of the random effects

Based on AIC, the selected compound symmetry model (CSM) was *CS.Rhe*. For MTM, the best one was *MT.Rhe*, and, among the RRM, the best one was *RR.2.Rhe* (Table 1). Hence, these three models, were used to estimate the variance components and to predict the genotypic values. Considering all models of this study, the RRM was a best fitted model comparing to MTM and CSM, as stated by AIC. In addition, according to the LRT, significant genotype effects were detected by all models (CSM, MTM, and RRM) and GEI effects was detected by CSM (Table 1).

Table 1. Model fitted, Akaike information criterion (AIC), and the likelihood ratio test (LRT) for genotypic and GEI effects, between parentheses, for the GY trait assessed in 84 interpopulational hybrids evaluated in four environments

Model[a]	k[b]	AIC	LRT
CS.Rho	3	14904.06	72.16** (14.11**)
CS.Rhe	6	14895.57[#]	74.06** (11.92**)
MT.Rho	11	14888.15	211.74**
MT.Rhe	14	14881.71[#]	203.61**
RR.1.Rho	2	14764.32	82.95**
RR.2.Rho	4	14751.88	91.17**
RR.3.Rho	7	14754.96	92.63**
RR.4.Rho	11	14736.76	105.73**

RR.1.Rhe	5	14753.98	81.08**
RR.2.Rhe	7	14738.36#	90.89**
RR.3.Rhe	10	14741.68	92.23**
RR.4.Rhe	14	14748.26	92.94**

[a] Compound symmetry models were referred to as *CS.R_*, multiple-trait models were referred to as *MT.R_*, and random regression models were referred as *RR.O.R_*; where *R_* may assumed homogeneous (Rho) or heterogenous (Rhe) residual variance structures, and *O* represents the orthogonal polynomial order fitted to the genotypic effect. [b] Number of parameters; #indicated the selected model by AIC (lower AIC); ** Significant at 1% of probability by the chi-square test. The null hypothesis tested was that random effects did not differ from zero.

Variance components and genetic parameters

Single estimates of genotypic and GEI variances were given by CSM (Table 2). Conversely, MTM and RRM presented one estimative of genetic variances for each environment but was not obtained any GEI variance estimation (Table 2). Regarding to MTM and RRM, specifically, the highest values, in both models, of genotypic variance ($\hat{\sigma}_g^2$) were detected in environments E1 and E2, respectively. The environment E4 presented the lowest values for both models, while the environment E3 presented the highest values of residual variance ($\hat{\sigma}_e^2$) (Table 2).

Table 2. Variance components and genetic parameters by the three models: compound symmetry (CSM), multiple-trait (MTM), and random regression (RRM).

Component/Parameter	CSM	MTM	RRM
$\hat{\sigma}_{g1}^2$	324884.60	785802.30	467421.77
$\hat{\sigma}_{g2}^2$		537714.80	533106.91

$\hat{\sigma}^2_{g3}$		313339.20	391987.53
$\hat{\sigma}^2_{g4}$		250269.40	226533.74
$\hat{\sigma}^2_{e1}$	971531.00	852670.90	1054350.00
$\hat{\sigma}^2_{e2}$	743312.90	752053.70	753868.00
$\hat{\sigma}^2_{e3}$	1092919.30	1125631.50	1208480.00
$\hat{\sigma}^2_{e4}$	678742.60	690943.60	702445.00
$\hat{\sigma}^2_{ge}$	124696.50	-	-
h^2_1	0.23	0.48	0.31
h^2_2	0.27	0.42	0.41
h^2_3	0.21	0.22	0.24
h^2_4	0.29	0.27	0.24
c^2_1	0.11	-	-
c^2_2	0.14	-	-
c^2_3	0.10	-	-
c^2_4	0.16	-	-
$r_{\hat{g}g_1}$	0.88	0.89	0.91
$r_{\hat{g}g_2}$		0.91	0.90
$r_{\hat{g}g_3}$		0.82	0.91
$r_{\hat{g}g_4}$		0.83	0.81
CV_{e1}	13.04	12.21	13.58
CV_{e2}	11.01	11.07	11.09
CV_{e3}	14.53	14.74	15.28
CV_{e4}	14.74	14.87	14.99
r_{gloc}	0.72	-	-

μ_1	7560.18
μ_2	7832.41
μ_3	7196.73
μ_4	5590.70

$\hat{\sigma}^2_{gi}$: genotypic variance in the environment *i*; $\hat{\sigma}^2_{ei}$: residual variance in the environment *i*; $\hat{\sigma}^2_{ge}$: genotype-by-environment interaction variance; h^2_i: heritability in the environment *i*; c^2_i: coefficient of determination of genotype-by-environment effect in the environment *i*; $r_{\hat{g}g_i}$: selective accuracy in environment *i*; CV_{ei}: experimental coefficient of variation in the environment *i*; r_{gloc}: genotypic correlation among environments; μ_i: phenotypic mean for the GY trait in the environment *i*.

Heritabilities (h^2) estimates did not follow any pattern. By CSM the estimates of heritabilities varied from 0.21 (E3) to 0.29 (E4) (Table 2). By the MTM and RRM similar results were obtained (Table 2). Regarding selective accuracy ($r_{\hat{g}g}$), the CSM estimated a single value for all environments (0.88), while MTM and RRM estimated selective accuracies for each environment (Tables 2 and S1.3). The environment E4 was the only one where CSM presented higher selective accuracy comparing to the other models (Tables 2 and S1.3). On the other environments, the selective accuracies of MTM or RRM were slightly superior over the CSM (Tables 2 and S1.3). For CSM, the genotypic correlation performance among environments (r_{gloc}) was 0.72.

Reaction norms

In Fig 1, the reaction norms were adjusted through Legendre polynomials of degree 2, which confirms the presence of the GEI effect, as the ranking changes over the environmental gradient (Table S1.4) [30]. When the genotypes vary their performances

over the environments, it is a complex interaction, since the reaction norms cross and ranking changes among the four environments.

Fig 1: Reaction norms of the 84 genotypes. The red lines refer to the five best and worst genotypes performances over the environmental gradient.

Selection gain

The highest predict gains were observed in environments E1 and E2, for both three models (Table 3). MTM obtained the highest gains, in all scenarios, being five selected genotypes, in environment E1, the highest gain overall (25.54%). As expected, gain decreases as the number of selected genotypes increases, i.e., the scenario of 20 selected genotypes, by CSM, predicted gain of 6.45% was obtained. The SG from RRM were slightly higher than CSM, which, in some scenarios and environments, has a higher predict gain but is overall below the MTM and RRM predicted gains (Fig S1.5).

Table 3. Predicted selection gains (in percentage) by the three models: compound symmetry (CSM), multiple-trait (MTM) and random regression (RRM).

Selection intensity	E1	E2	E3	E4
		CSM		
5	21.79	20.07	13.11	13.49
10	15.65	14.48	8.95	10.76
15	13.17	11.02	7.82	8.92
20	11.43	9.33	6.45	6.98

		MTM		
5	25.54	20.29	13.91	15.69
10	19.29	15.35	10.78	12.26
15	16.05	12.38	8.92	10.50
20	13.75	10.56	7.74	9.12
		RRM		
5	25.06	20.29	13.00	14.74
10	18.86	15.35	9.78	11.45
15	14.86	12.29	8.43	10.27
20	12.95	10.48	7.16	8.90

Discussion

~~Akaike information criterion (AIC) is among the most adopted statistical criterion for model selection, ranking models based on efficiency [31]. In addition, in RRM, when selecting the polynomial order, AIC has been widely used [31]. Moreover, many studies adopted AIC as criterion for model selection [32–34].~~ Based on AIC, the RRM was superior over MTM and CSM, being a best fitted model in our study.~~ The LRT is based on the difference between the maximum likelihood under the null and under the alternative hypothesis (i.e. when the referred effect is not considered).~~ Our results indicated that the genotypic and GEI effects were significant, ~~by LRT.~~

The MTM and RRM used in this study allows modeling the genetic and residual covariance structures, aiming to better understand these effects. The adequate modeling of genotypic and residual effects is a way to mitigate the GEI effects and maximize the selective accuracy [35–37]. Studies with maize, potato, and wheat achieve more than 5% of gain in the selective accuracy when modelled the GEI effect [38]. The heterogeneous

residual variance structure was the best fitted in all models (*CS.Rhe*, *MT.Rhe*, and *RR.2.Rhe*), being models with this residual structure stated by some studies in annual crops [33,39,40]. In addition, the RRM best fitted was *RR.2.Rhe* which is the Legendre polynomials of second order for the genetic effect.

After selection of the best fitted model for the three methods studied, the estimated genetic and non-genetic parameters were compared. The estimated residual variance presented greater disparity, in all environments. As it presented by MTM, where the environment E3 presented values 60% higher than those from the environment E4 (Table 2). Similar results were obtained by CSM and RRM (Table 2). This discrepancy reinforces the results found by AIC and supports the choice for the models with heterogeneous residual variance.

The genotypic correlation among environments, given by CSM, below 0.75 demonstrate the necessity of more robust analysis for the genetic selection [7]. Moreover, the CSM assumes a constant genetic variances and correlations between environments equal to one, what are not a prerequisite in MTM and RRM [10]. In this way, when complex GEI occurs, models that incorporate precise variance structures should be preferred, such as MTM and RRM [7], which demonstrates the fragility of CSM.

The evaluation of GEI can be developed through several methods [14]. Alves et al. [6] believe that new methodologies based on mixed models achieve most effectivity in the genetic selection, such as Factor Analytic Multiplicative Mixed Models [41] and RRM. The most complex and parameterized model, i.e. MTM, estimates each random effect for each environment, simultaneously. Additionally, covariances between each pair of environments are also estimated. RRM also estimates individual variance components for each environment and covariance between each pair of environments. It has been applied to capture the change of a trait continually over growth stages (in perennial

species) or environmental gradients (in MET), with fewer parameters to be estimated, compared to MTM [8,11]. In our study, the MTM and RRM estimated 14 and 7 parameters, respectively, whereas the CSM presented 6 parameters. The parsimony (i.e. lower number of parameters) is the only advantage of CSM over MTM and RRM.

The CSM allowed estimate the GEI variance and, its division by the phenotypic variance is the coefficient of determination of GEI effects. In this study, CSM, indicated complex GEI effect (i.e. significant GEI effects by LRT), as well as in RRM (i.e. polynomial order 2). In the random regression context, the GEI effects can be detected through reaction norms [30]. The GEI occurs when the reaction norms intersect, diverge, or converge [30]. Our study verified intersections of reaction norms through environmental gradient for many genotypes. Also, it can be noted in some relevant studies with elephant-grass and eucalyptus, indicates the presence of complex GEI in the reaction norms [6,42]. In the MTM, the GEI effects cannot be direct estimated, since these effects are confounded with the genetic effects ($\hat{\sigma}^2_{g_i} = \hat{\sigma}^2_{g_i} + \hat{\sigma}^2_{ge_i}$). However, if the genotype ranking differs among environments, the complex GEI occurs. Volpato et al. [43] demonstrate the presence of this effect in genotype ranking, by MTM.

As MTM presents an unstructured matrix for the random effects, it is able to estimate variance components per environment and covariance between pair of environments, being considered a complete model. Although the algorithms AI (average information) or EM (expectation-maximization) are widely applied in the estimation process, they face some issues regarding convergence for unstructured matrix, in MTM [8]. Conversely, the estimation process of random effects, in RRM, requests a reduced structure and fewer parameters, comparing to MTM [6,8,11]. Our study presents satisfactory results for both models, however, in case of larger number of environments,

MTM would become prohibitive. In addition, RRM has the capability to estimate continuous variance function through the observed genotypic effects matrix.

The heritability values are more accurate in MTM and RRM than in CSM, because these models consider heterogeneity of genetic variances across environments [6]. This characteristic carries out to more accurate, and higher values of heritabilities were estimated by MTM and RRM in this study. Regarding heritability estimation, the superiority of RRM over CSM is also reported by Alves et al. [6]. This parameter can be affected by any change in the variance components, i.e. genetic or residual variances [44]. In addition, this parameter is of interest in any breeding program, since the genotypes selection will consider their predicted values, based on heritabilities estimates [45].

In genetic evaluation context, the selective accuracy is the most important statistical parameter, as mentioned by Henderson [46]. It is able to provide the right ranking of genotypes [46]. Also, this parameter is defined as the correlation between predicted and real genotypic values [47]. In MTM, the increase of accuracy is proportional to the difference between genetic and environmental correlations of the trait [48], and one of the advantages of MTM is providing the higher selective accuracy [8,49]. Resende and Duarte [15] state that accuracy is a way to report about the experimental quality, since this parameter consider residual and genetic variances. According to Resende and Duarte [15], in MTM, a very high selective accuracy was estimated for the environment E2 (Table 2). On the other hand, in RRM, the environment E4 presented high selective accuracy, while the other environments presented very high accuracies (Table 2).

Regarding to selection gain, and considering the above-mentioned parameters, MTM and RRM presented slightly superiority in gains, as they estimated one genotypic value per environment and considers the genotypic correlation between pair of

environments. These methods reach 25% of gain when selecting 5 genotypes (Table 3). The environments E1 and E2 stood out for gains, which were over 5% for other environments, when comparing the same selection intensity (Table 3). Conversely, for CSM, the worst environment (E3) revealed low gains from 6.45% to 13.11%, when selected 5 to 20 genotypes, respectively. This difference among models can be explained by the variance component estimates, which infer a higher heritability (h^2), for MTM and RRM, based on the characteristic of the environment (biotic and abiotic stress) and experimental quality (high selective accuracy and experimental coefficient of variation) [5,15]. It is important to highlight that the selective accuracies of CSM, which were, in some cases, even higher than MTM and RRM. This can be interpreted as a negative aspect of CSM, which does not consider the difference between genotypic variance ($\hat{\sigma}_g^2$) in each environment, since it considers only the overall genotypic variance and GEI variance, providing misestimated accuracies. For instance, the lower experimental quality for environment E4, compared to the others (E1, E2 and E3) in this study, is confirmed by experimental coefficient of variation and selective accuracy for MTM and RRM (Table 2), which is not detected in CSM estimates.

The difference among the genotypes ranking, in all models, is due the GEI effect, which affects the performance of the genotypes in different environments [14]. As mentioned by van Eeuwijk et al. [30], when genes express differently over environments, the GEI will occur. So, the different rankings (Table S1.4) are another proof of GEI effect in our study, for all models. Finally, the presence of some inter-population hybrids is observed in the ranking, figuring out among the top commercial hybrids, which reveals the high potential of these genotypes (Table S1.4). The breeder can focus on the parental hybrids of these genetic materials, and, in which environments they performed better, to recommend basing on each one environment individually.

The best models to adopt are the MTM and RRM, as they allowed to estimate the genotypic variance ($\hat{\sigma}_g^2$) per environment. Nevertheless, the heterogeneous structure of CSM provides residual variance ($\hat{\sigma}_e^2$) per environments. But, the absence of genetic variance estimates per environment prevents CSM from providing genetic correlation among environments, which can be a disadvantage comparing to MTM and RRM. Even so, all these methods provide estimates of variance components and genetic parameters per environment. For maize breeding, GEI is a relevant effect and the development of genetic materials must consider it [4,5].

Conclusion

The RRM was superior over MTM and CSM by AIC for dealing with MET, being a best fitted model. Although MTM and RRM present detailed variance components and genetic parameter estimates, based on the selective accuracies and heritabilities estimates, the RRM was slightly superior. These models have the capability to mitigate all random effects of the model. RRM also offers reliable results and can reduce the number of parameters, besides the convergence issues. In this case, to solve a potential overflow issue of estimates, in MTM, the RRM is superior. In practical terms, the MTM presented slightly higher gains, what can be justified by the computational efforts required by the model. It becomes prohibitive when there is higher number of evaluated environments. Finally, interpopulational hybrids present better performances over some commercial hybrids, in some environments.

References

1. USDA. Economic Research Service. 2019.

2. Resende MDV. Genética quantitativa e de populações. Suprema, Visconde do Rio Branco. 1st ed. 2015; 463.

3. Marchal A, Schlichting CD, Gobin R, Balandier P, Millier F, Muñoz F, et al. Genotype by environment interactions in forest tree breeding: review of methodology and perspectives on research and application. Plant Genome. 2019;13: 281–288. doi:10.1017/S0021859605005587

4. Bernardo R. Breeding for Quantitative Traits in Plants. 2nd ed. Woodbury, Minnesota: Stemma Press; 2010.

5. Lima RO de, Borém A. Melhoramento de Milho. Viçosa - MG: Editora UFV; 2018.

6. Alves RS, de Resende MDV, Azevedo CF, Silva FF e, Rocha JR do AS de C, Nunes ACP, et al. Optimization of Eucalyptus breeding through random regression models allowing for reaction norms in response to environmental gradients. Tree Genet Genomes. 2020;16: 38. doi:10.1007/s11295-020-01431-5

7. Resende MDV de. Matemática e estatística na análise de experimentos e no melhoramento genético. Embrapa Florestas, Colombo; 2007.

8. Resende MD V, Silva FF, Azevedo CF. Estatística matemática, biométrica e computacional: modelos mistos, multivariados, categóricos e generalizados (REML/BLUP), inferência bayesiana, regressão aleatória, seleção genômica, QTL-QWAS, estatística espacial e temporal, competição, sobrevivência. Suprema, Visconde do Rio Branco. 2014;1: 881.

9. Sun J, Rutkoski JE, Poland JA, Crossa J, Jannink JL, Sorrells ME. Multitrait, random regression, or simple repeatability model in high-throughput phenotyping data improve genomic prediction for wheat grain yield. Plant Genome. 2017;10. doi:10.3835/plantgenome2016.11.0111

10. Meyer K. Random regressions to model phenotypic variation in monthly weights of Australian beef cows. Livest Prod Sci. 2000;65: 19–38. doi:10.1016/S0301-6226(99)00183-9

11. Rocha JR do AS de C, Marçal T de S, Salvador FV, da Silva AC, Machado JC, Carneiro PCS. Genetic insights into elephantgrass persistence for bioenergy purpose. PLoS One. 2018;13: 1–16. doi:10.1371/journal.pone.0203818

12. Kirkpatrick M, Heckman N. A quantitative genetic model for growth, shape, reaction norms, and other infinite-dimensional characters. J Math Biol. 1989;27: 429–450. doi:10.1007/BF00290638

13. Meyer K. Modeling “repeated” records : Covariance functions and random regression models to analyse animal breeding data. Proceedings of the 6th World Congress on Genetics Applied to Livestock Production. 1998. pp. 517–520.

14. Li Y, Suontama M, Burdon RD, Dungey HS. Genotype by environment interactions in forest tree breeding: review of methodology and perspectives on research and application. Tree Genet Genomes. 2017;13. doi:10.1007/s11295-017-1144-x

15. Resende MDV De, Duarte JB. Precisão e controle de qualidade em experimentos de avaliação de cultivares. Pesqui Agropecuária Trop. 2007;37: 182–194. doi:10.5216/pat.v37i3.1867

16. Martin JGA, Nussey DH, Wilson AJ, Réale D. Measuring individual differences in reaction norms in field and experimental studies: a power analysis of random regression models. Methods Ecol Evol. 2011;2: 362–374. doi:10.1111/j.2041-210X.2010.00084.x

17. Brommer JE, Kontiainen P, Pietiäinen H. Selection on plasticity of seasonal life-history traits using random regression mixed model analysis. Ecol Evol. 2012;2:

695–704. doi:10.1002/ece3.60

18. Mota RR, Tempelman RJ, Lopes PS, Aguilar I, Silva FF, Cardoso FF. Genotype by environment interaction for tick resistance of Hereford and Braford beef cattle using reaction norm models. Genet Sel Evol. 2016;48: 3. doi:10.1186/s12711-015-0178-5

19. Arnold PA, Kruuk LEB, Nicotra AB. How to analyse plant phenotypic plasticity in response to a changing climate. New Phytol. 2019;222: 1235–1241. doi:10.1111/nph.15656

20. Alvares CA, Stape JL, Sentelhas PC, de Moraes Gonçalves JL, Sparovek G. Köppen's climate classification map for Brazil. Meteorol Zeitschrift. 2013;22: 711–728. doi:10.1127/0941-2948/2013/0507

21. Cruz JC, Karam D, Monteiro MAR, Magalhães PC. A cultura do milho. Sete Lagoas, MG: Embrapa Milho e Sorgo; 2008.

22. Gilmour AR, Gogel BJ, Cullis BR, Welham Sj, Thompson R. ASReml user guide release 4.1 structural specification. Hemel hempstead VSN Int ltd. 2015.

23. Schaeffer LR. Random Regression Models. 2016; 171.

24. Akaike H. A new look at the statistical model identification. Selected Papers of Hirotugu Akaike. Springer; 1974. pp. 215–222.

25. Wilks SS. The Large-Sample Distribution of the Likelihood Ratio for Testing Composite Hypotheses. Ann Math Stat. 1938;9: 60–62. Available: https://www.jstor.org/stable/pdf/2957648.pdf

26. Kirkpatrick M, Lofsvold D, Bulmer M. Analysis of the inheritance, selection and evolution of growth trajectories. Genetics. 1990;124: 979–993.

27. Resende MDV. Software Selegen-REML/BLUP: a useful tool for plant breeding. Crop Breed Appl Biotechnol. 2016;16: 330–339. doi:10.1590/1984

28. R Core Team. R: A language and environment for statistical computing. Vienna, Austria: R Foundation for Statistical Computing; 2019.

29. Butler DG, Cullis BR, Gilmour AR, Gogel BJ. ASReml-R reference manual (version 3). The State of Queensland, Department of Primary Industries and Fisheries: Brisbane, Qld. 2009.

30. Van Eeuwijk FA, Bustos-Korts D V., Malosetti M. What should students in plant breeding know about the statistical aspects of genotype × Environment interactions? Crop Sci. 2016;56: 2119–2140. doi:10.2135/cropsci2015.06.0375

31. Corrales JD, Munilla S, Cantet RJC. Polynomial order selection in random regression models via penalizing adaptively the likelihood. J Anim Breed Genet. 2015;132: 281–288. doi:10.1111/jbg.12130

32. Faveri J De, Verbyla AP, Pitchford WS, Venkatanagappa S, Cullis BR. Statistical methods for analysis of multi-harvest data from perennial pasture variety selection trials. Crop Pasture Sci. 2015;66: 947. doi:10.1071/CP14312

33. Melo VL de, Marçal T de S, Rocha JRAS de C, dos Anjos RSR, Carneiro PCS, Carneiro JE de S. Modeling (co)variance structures for genetic and non-genetic effects in the selection of common bean progenies. Euphytica. 2020;216: 77. doi:10.1007/s10681-020-02607-9

34. Abrantes KKB, Paiva LM, Almeida RG de, Urbano E, Ferreira AD, Mazucheli J. Modeling the individual height and volume of two integrated crop-livestock-forest systems of *Eucalyptus* spp. in the Brazilian Savannah. Acta Sci Agron. 2019;41: 42626. doi:10.4025/actasciagron.v41i1.42626

35. Smith A, Cullis B, Thompson R. Analyzing Variety by Environment Data Using Multiplicative Mixed Models and Adjustments for Spatial Field Trend. Biometrics. 2001;57: 1138–1147. doi:10.1111/j.0006-341X.2001.01138.x

36. Smith AB, Cullis BR, Thompson R. The analysis of crop cultivar breeding and evaluation trials: An overview of current mixed model approaches. J Agric Sci. 2005;143: 449–462. doi:10.1017/S0021859605005587
37. Smith AB, Ganesalingam A, Kuchel H, Cullis BR. Factor analytic mixed models for the provision of grower information from national crop variety testing programs. Theor Appl Genet. 2015;128: 55–72. doi:10.1007/s00122-014-2412-x
38. Burgueño J, Crossa J, Cotes JM, Vicente FS, Das B. Prediction Assessment of Linear Mixed Models for Multienvironment Trials. Crop Sci. 2011;51: 944–954. doi:10.2135/cropsci2010.07.0403
39. Rocha JR do AS de C, Nunes KV, Carneiro ALN, Marçal TDS, Salvador FV, Carneiro PCS, et al. Selection of Superior Inbred Progenies toward the Common Bean Ideotype. Agron J. 2019;1181–1189: 1181–1189. doi:10.2134/agronj2018.12.0761
40. So YS, Edwards J. Predictive ability assessment of linear mixed models in multienvironment trials in corn. Crop Sci. 2011;51: 542–552. doi:10.2135/cropsci2010.06.0338
41. Resende MDV de, Thompson R. Factor analytic multiplicative mixed models in the analysis of multiple experiments. Rev Matemática e Estatística. 2004;22: 31–52.
42. Rocha VD, Tiago PV, Tiago AV, Pedri ECM, Cardoso ES, Rossi AAB. Genetic diversity among Hymenaea courbaril L. genotypes naturally occurring in the north of Mato Grosso State, Brazil. Genet Mol Res. 2017;16. doi:10.4238/gmr16039706
43. Volpato L, Alves RS, Teodoro PE, De Resende MDV, Nascimento M, Nascimento ACC, et al. Multi-trait multi-environment models in the genetic

selection of segregating soybean progeny. PLoS One. 2019;14: 1–22. doi:10.1371/journal.pone.0215315

44. Batista CM, Freitas MLM, de Moraes MA, Zanatto ACS. Estimates of genetic parameters and variability in provenances and progeny of Handroanthus velossoi. Pesqui Florest Bras. 2012;32: 269–276. doi:0.4336/2012.pfb.32.71.269

45. Maia MCC, Resende MDV de, Paiva JR de, Cavalcanti JJV, Barros L de M. Seleção simultânea para produção , adaptabilidade e estabilidade genotípicas em clones de cajueiro , via modelos mistos 1. Pesqui Agropecuária Trop. 2009;39: 43–50.

46. Henderson CR. Applications of linear models in animal breeding. University of Guelph Guelph; 1984.

47. Van Vleck LD, Pollak EJ, Oltenacu EAB. Genetics for the Animal Sciences. New York: W.H. Freeman and Company; 1987.

48. Thompson R, Meyer K. A review of theoretical aspects in the estimation of breeding values for multi-trait selection. Livest Prod Sci. 1986;15: 299–313. doi:10.1016/0301-6226(86)90071-0

49. Okeke UG, Akdemir D, Rabbi I, Kulakow P, Jannink JL. Accuracies of univariate and multivariate genomic prediction models in African cassava. Genet Sel Evol. 2017;49: 1–10. doi:10.1186/s12711-017-0361-y

Supporting information

Table S1.1. Location of environments, with the corresponding geographic coordinates and altitudes.

Table S1.2. Complete diallel scheme involving thirteen F_2 population (used as parents) and their respectively crosses (H).

Table S1.3. Selective accuracy by each genotype, per environment, for: Compound Symmetry (CSM, Multiple-Trait (MTM) and Random Regression (RRM) models.

Table S1.4. Ranking for compound symmetry (STM), multiple-trait (MTM) and random regression (MTM) models, in each environment (E1 to E4). Until the dashed line are the top ten hybrids, and it is highlighted the inter-population hybrids into it.

Fig S1.5. Coincidence index, for all environments in all methodologies. The compound symmetry (CSM), multiple-trait (MTM) and random regression (MTM) models, are followed by each environment (_1, _2, _3 and _4). The letters A, B, C and D correspond to each scenario (5, 10, 15, and 20, respectively).

Response letter

Dear Dr. Diogo Borges Provete, we are sending the revised version of the manuscript PONE-D-20-15130 - Multiple-trait, random regression, and compound symmetry models for analysis of multi-environment trials in maize breeding.

We would like to thank you and the reviewer for the excellent contributions to improvement of this manuscript. The changes, based on the questions and comments, were highlighted in blue (the same color of the answers in this letter).

Below we respond individually each comment and we are available for any questions.

Reviewer #1:

"…. Second the reviewer that this paper might have a large impact, but authors must do a much better job in accomodating the questions into the structure of the text by making it more concise and straightforward. Your main goal is relevant by itself, so avoid trying to fit in other parallel topics into the manuscript. I have provided a fully commented PDF attached. Below I listed my major concerns:"

1. Line 148: "Degrees of fit?"

Response: In the random regression analyses, the degrees of fit are given by the number of environments (or repeated measures)

2. "no need to state the formula of AIC, which is widely used. Question: why didn't you use AICc instead?" …. Table 1 (line 236) "provide deltaAICc instead of raw AIC values, which are meaningless"

Response: The AIC and BIC are the standard criteria for models choice (Cavanaugh & Neath, 2019; Neath & Cavanaugh, 2012). Models with the same fixed terms are readily

compared by these criteria (Verbyla, 2019). The AIC and BIC have been widely used in model selection in plant breeding data analyses (Alves et al., 2020; Melo et al., 2020).

We appreciate the suggestion, and we already cut off the AIC formula from the manuscript. We did not use the AICc because the *n* was large enough, 985. According to Brewer et al. (2016), the AICc is a correction in case of a small *n*, providing a stronger penalty than AIC for smaller sample. Although, Burnham and Anderson (2004) infers that AICc → AIC as n $\rightarrow \infty$.

3. “The abstract needs to be fully revised. It doesn't state clearly the context of the study and provide a good summary of the results and conclusions?”

We have re-written the abstract.

4. “Most part of the Results is about comparing the parameters estimated in each of the three models. Since it seems important for the paper, why don't you also provide an estimate of uncertainty around each parameter? Consider fitting models under a Bayesian framework.”

As commented by the other reviewer, this article is already carrying a lot of information, in fact, would be enriching to provide an uncertainty measure about the parameter, but we believed we have enough information to discuss about the parameters. The use of Bayesian inference, with no prior information, conducts to the same results (Gianola & Fernando, 1986). In addition, the implementation of these models under Bayesian framework requires more computational efforts (processing capacity and time). The idea of fit models under Bayesian framework is very good and we will consider in a future work.

5. “The discussion must be almost entirely re-written and restructured. It barely cites other papers in the fiels, even when there're a couple of reviews on the topic. Most of paragraphs

have only one or two citations, this is not good practice in scientific writing. You have to promote a dialogue between your results and previous studies.”

We re-wrote and restructured the discussion as required.

6. “Most importantly, English language grammar and writing style need to be carefully revised throughout the whole manuscript. There're sentences that are really hard to understand, the reviewer also complained about it. Some parts of the text are also too much verbose, especially in the Results, try to reduce them to make it straightforward.”

We made these changes. See the Results.

Thank you very much for the review and for the excellent contributions to improvement of this manuscript.

Reviewer #2:

Coelho et al present a detailed multi-environmental trial analysis of 84 maize hybrids. Their goal, as stated in the abstract, is to better understand the extent to which GEI interactions might be relevant to maize breeding programs. In order to do so, the authors use a model-fitting approach, comparing the model fit of three main model classes of widespread use (CSM, MTM and RRM). Overall, the goal of this paper is clearly laid out, and the set of analyses is properly aimed at answering this key aspect of genetic variation patterns. Likewise, the amount of effort required to produce such dataset is commendable. Having said that, this version of the manuscript also has some significant shortcomings, especially with regards to organization/clarity and in placing the work in a greater context. A significant rewrite is likely required. In terms of organization and clarity, a substantive issue is the fact that the manuscript still tries to tackle too many issues at the same time, making it hard to follow. As made clear in the introduction, the

goal of the manuscript is straightforward: compare these three model classes and infer the practical consequences of choosing one over the other (in terms of GEI). While the goal is clear, I feel that the manuscript falls short of such a goal, especially with regards to the interpretation of the results.

1. “While the manuscript reports detailed estimates of genetic variance, it does not address the ways in which all of these estimates have very different interpretations. For example, the estimate of GEI variance in the CSM model has no clear relationship to any of the parameters in the remaining models. However, it does have a potentially useful and clear interpretation. It can be not only interpreted as the portion of the total phenotypic variance that is due to GEI interactions, but can also be compared across traits or species, becoming therefore particularly useful. The question that emerges is then, what is the practical significance of the parameters in MTM and RRM models? To what extent can they provide a intuitive understanding of the GEI interaction?”

Response: We made the changes (see Discussion, specifically lines 326-333).

“In the random regression context, the GEI effects are detected through reaction norms (Van Eeuwijk et al., 2016). The GEI occurs when the reaction norms intersect, diverge, or converge (Van Eeuwijk et al., 2016). In the multiple-trait models, the GEI effects can not be estimated, since these effects are confounded with the genetic effects ($\hat{\sigma}^2_{g_i} = \hat{\sigma}^2_{g_i} + \hat{\sigma}^2_{ge_i}$). However, if the genotype ranking differs among environments, the complex GEI occurs.”

2. “Instead, right now the manuscript goes back and forth between different topics. As such, the manuscript would greatly benefit from some streamlining. The measures of

selective accuracy and selection gain are, for example, rather tangential to the main goal. Selective accuracy, in particular, is purely a theoretical expectation and follows directly from the heritability estimates. Essentially, the higher the heritability of a trait, the lower the prediction error variance. Perhaps eliminating some of these tangential aspects would help clarify the main thread. As is, the manuscript is an ensemble of variance estimates and the discussion does not help clarify their interpretation."

Response: The selective accuracy is the most important parameter for the genetic recommendation in plant breeding (Resende & Duarte, 2007). This parameter corresponds to the correlation between predict and true genetic values (Resende & Duarte, 2007) and it is a reliability measure.

3. "I would also add that the manuscript would greatly benefit from clearer figures. Most notably, Figure 1 seems to suggest that most environments have similar rankings of maize hybrids. While some hybrids have lower or higher yield across environments, their ranking seems to remain essentially the same. Instead, the remaining of the manuscript argues for the opposite of that. Given that changes in ranking have different implications than pure increases in the yield spread, clarifying this aspect of the manuscript might be particularly important. If all the hybrids that perform well in a single environment, perform (comparatively) as well in others, then GEI interactions (while still capable of generating variance) are of less importance for practical purposes, since all one would need to do is to select the ones with the highest yield at a single environment."

Response: In fact, in this study, the best genotypes in one environment are also the best in the others. However, considering all genotypes in all environments the GEI is very clear, since the reaction norms cross. We cleared the figure. Similar results were found by Alves et al. (2020) evaluating *Eucalyptus* clones, for the Pylodin penetration trait.

Besides that, these authors verified that for traits with higher coefficients of determination of GEI variances, the genotypes raking varies more along the environmental gradient.

4. “Finally, I would emphasize that ‘less is more’ in this case. The more clearly defined the manuscript becomes, the higher the impact it will have in its field.”

“Minor comment: - The colors in most figures are hard to read and follow.”

Response: We re-made the Results and Discuss almost entirely. (See Results and Discussion)

Thank you very much for the review and for the excellent contributions to improvement of this manuscript.

Alves, R. S., Resende, M. D. V., Azevedo, C. F., Silva, F. F., Rocha, J. R. do A. S. de C., Nunes, A. C. P., Carneiro, A. P. S., & Santos, G. A. (2020). Optimization of Eucalyptus breeding through random regression models allowing for reaction norms in response to environmental gradients. *Tree Genetics & Genomes*, *16*(2), 38. https://doi.org/10.1007/s11295-020-01431-5

Brewer, M. J., Butler, A., & Cooksley, S. L. (2016). The relative performance of AIC, AIC C and BIC in the presence of unobserved heterogeneity. *Methods in Ecology and Evolution*, *7*(6), 679–692. https://doi.org/10.1111/2041-210X.12541

Burnham, K. P., & Anderson, D. R. (2004). Multimodel inference: understanding AIC and BIC in model selection. *Sociological Methods and Research*, *33*, 261–304.

Cavanaugh, J. E., & Neath, A. A. (2019). The Akaike information criterion : Background , derivation , properties , application , interpretation , and refinements. *Wiley*

Computational Statistics, *11*(January), 1–11. https://doi.org/10.1002/wics.1460

Gianola, D., & Fernando, R. L. (1986). Bayesian methods in animal breeding theory. *Journal of Animal Science*, *63*(1), 217–244.

Melo, V. L. de, Marçal, T. de S., Rocha, J. R. A. S. de C., dos Anjos, R. S. R., Carneiro, P. C. S., & Carneiro, J. E. de S. (2020). Modeling (co)variance structures for genetic and non-genetic effects in the selection of common bean progenies. *Euphytica*, *216*(5), 77. https://doi.org/10.1007/s10681-020-02607-9

Neath, A. A., & Cavanaugh, J. E. (2012). The Bayesian information criterion: background, derivation, and applications. *Wiley Interdisciplinary Reviews: Computational Statistics*, *4*(2), 199–203. https://doi.org/10.1002/wics.199

Resende, M. D. V. De, & Duarte, J. B. (2007). Precisão e controle de qualidade em experimentos de avaliação de cultivares. *Pesquisa Agropecuária Tropical*, *37*(3), 182–194. https://doi.org/10.5216/pat.v37i3.1867

Van Eeuwijk, F. A., Bustos-Korts, D. V., & Malosetti, M. (2016). What should students in plant breeding know about the statistical aspects of genotype × Environment interactions? *Crop Science*, *56*(5), 2119–2140. https://doi.org/10.2135/cropsci2015.06.0375

Verbyla, A. P. (2019). A note on model selection using information criteria for general linear models estimated using REML. *Australian and New Zealand Journal of Statistics*, *61*(1), 39–50. https://doi.org/10.1111/anzs.12254
