# Supplementary material for: Multiple-trait, random regression, and compound symmetry models for analyzing multi-environment trials in maize breeding
Source: PLoS One. 2020 Nov 20;15(11):e0242705. doi: 10.1371/journal.pone.0242705 (PMC7678961; doi:10.1371/journal.pone.0242705)
Supplement: Attachment — Submitted filename: Response letter.docx [file pone.0242705.s004.docx]

**Response letter**

Dear Dr. Diogo Borges Provete, we are sending the revised version of the manuscript PONE-D-20-15130 - Multiple-trait, random regression, and compound symmetry models for analysis of multi-environment trials in maize breeding.

We would like to thank you and the reviewer for the excellent contributions to improvement of this manuscript. The changes, based on the questions and comments, were highlighted in blue (the same color of the answers in this letter).

Below we respond individually each comment and we are available for any questions.

**Reviewer #1**:

“…. Second the reviewer that this paper might have a large impact, but authors must do a much better job in accomodating the questions into the structure of the text by making it more concise and straightforward. Your main goal is relevant by itself, so avoid trying to fit in other parallel topics into the manuscript. I have provided a fully commented PDF attached. Below I listed my major concerns:”

1. Line 148: “Degrees of fit?”

Response: In the random regression analyses, the degrees of fit are given by the number of environments (or repeated measures)

2. “no need to state the formula of AIC, which is widely used. Question: why didn't you use AICc instead?” …. Table 1 (line 236) “provide deltaAICc instead of raw AIC values, which are meaningless”

Response: The AIC and BIC are the standard criteria for models choice (Cavanaugh & Neath, 2019; Neath & Cavanaugh, 2012). Models with the same fixed terms are readily compared by these criteria (Verbyla, 2019). The AIC and BIC have been widely used in model selection in plant breeding data analyses (Alves et al., 2020; Melo et al., 2020).

We appreciate the suggestion, and we already cut off the AIC formula from the manuscript. We did not use the AICc because the *n* was large enough, 985. According to Brewer et al. (2016), the AICc is a correction in case of a small *n*, providing a stronger penalty than AIC for smaller sample. Although, Burnham and Anderson (2004) infers that AICc $\to$ AIC as n $\to\infty$.

3. “The abstract needs to be fully revised. It doesn't state clearly the context of the study and provide a good summary of the results and conclusions?”

We have re-written the abstract.

4. “Most part of the Results is about comparing the parameters estimated in each of the three models. Since it seems important for the paper, why don't you also provide an estimate of uncertainty around each parameter? Consider fitting models under a Bayesian framework.”

As commented by the other reviewer, this article is already carrying a lot of information, in fact, would be enriching to provide an uncertainty measure about the parameter, but we believed we have enough information to discuss about the parameters. The use of Bayesian inference, with no prior information, conducts to the same results (Gianola & Fernando, 1986). In addition, the implementation of these models under Bayesian framework requires more computational efforts (processing capacity and time). The idea of fit models under Bayesian framework is very good and we will consider in a future work.

5. “The discussion must be almost entirely re-written and restructured. It barely cites other papers in the fiels, even when there're a couple of reviews on the topic. Most of paragraphs have only one or two citations, this is not good practice in scientific writing. You have to promote a dialogue between your results and previous studies.”

We re-wrote and restructured the discussion as required.

6. “Most importantly, English language grammar and writing style need to be carefully revised throughout the whole manuscript. There're sentences that are really hard to understand, the reviewer also complained about it. Some parts of the text are also too much verbose, especially in the Results, try to reduce them to make it straightforward.”

We made these changes. See the Results.

Thank you very much for the review and for the excellent contributions to improvement of this manuscript.

**Reviewer #2:**

Coelho et al present a detailed multi-environmental trial analysis of 84 maize hybrids. Their goal, as stated in the abstract, is to better understand the extent to which GEI interactions might be relevant to maize breeding programs. In order to do so, the authors use a model-fitting approach, comparing the model fit of three main model classes of widespread use (CSM, MTM and RRM). Overall, the goal of this paper is clearly laid out, and the set of analyses is properly aimed at answering this key aspect of genetic variation patterns. Likewise, the amount of effort required to produce such dataset is commendable. Having said that, this version of the manuscript also has some significant shortcomings, especially with regards to organization/clarity and in placing the work in a greater context. A significant rewrite is likely required. In terms of organization and clarity, a substantive issue is the fact that the manuscript still tries to tackle too many issues at the same time, making it hard to follow. As made clear in the introduction, the goal of the manuscript is straightforward: compare these three model classes and infer the practical consequences of choosing one over the other (in terms of GEI). While the goal is clear, I feel that the manuscript falls short of such a goal, especially with regards to the interpretation of the results.

1. “While the manuscript reports detailed estimates of genetic variance, it does not address the ways in which all of these estimates have very different interpretations. For example, the estimate of GEI variance in the CSM model has no clear relationship to any of the parameters in the remaining models. However, it does have a potentially useful and clear interpretation. It can be not only interpreted as the portion of the total phenotypic variance that is due to GEI interactions, but can also be compared across traits or species, becoming therefore particularly useful. The question that emerges is then, what is the practical significance of the parameters in MTM and RRM models? To what extent can they provide a intuitive understanding of the GEI interaction?”

Response: We made the changes (see Discussion, specifically lines 326-333).

“In the random regression context, the GEI effects are detected through reaction norms (Van Eeuwijk et al., 2016). The GEI occurs when the reaction norms intersect, diverge, or converge (Van Eeuwijk et al., 2016). In the multiple-trait models, the GEI effects can not be estimated, since these effects are confounded with the genetic effects ($\hat{\sigma}_{g_{i}}^{2}=\hat{\sigma}_{g_{i}}^{2}+\hat{\sigma}_{ge_{i}}^{2}$). However, if the genotype ranking differs among environments, the complex GEI occurs.”

2. “Instead, right now the manuscript goes back and forth between different topics. As such, the manuscript would greatly benefit from some streamlining. The measures of selective accuracy and selection gain are, for example, rather tangential to the main goal. Selective accuracy, in particular, is purely a theoretical expectation and follows directly

from the heritability estimates. Essentially, the higher the heritability of a trait, the lower the prediction error variance. Perhaps eliminating some of these tangential aspects would help clarify the main thread. As is, the manuscript is an ensemble of variance estimates and the discussion does not help clarify their interpretation.”

Response: The selective accuracy is the most important parameter for the genetic recommendation in plant breeding (Resende & Duarte, 2007). This parameter corresponds to the correlation between predict and true genetic values (Resende & Duarte, 2007) and it is a reliability measure.

3. “I would also add that the manuscript would greatly benefit from clearer figures. Most notably, Figure 1 seems to suggest that most environments have similar rankings of maize hybrids. While some hybrids have lower or higher yield across environments, their ranking seems to remain essentially the same. Instead, the remaining of the manuscript argues for the opposite of that. Given that changes in ranking have different implications than pure increases in the yield spread, clarifying this aspect of the manuscript might be particularly important. If all the hybrids that perform well in a single environment, perform (comparatively) as well in others, then GEI interactions (while still capable of

generating variance) are of less importance for practical purposes, since all one would need to do is to select the ones with the highest yield at a single environment.”

Response: In fact, in this study, the best genotypes in one environment are also the best in the others. However, considering all genotypes in all environments the GEI is very clear, since the reaction norms cross. We cleared the figure. Similar results were found by Alves et al. (2020) evaluating *Eucalyptus* clones, for the Pylodin penetration trait. Besides that, these authors verified that for traits with higher coefficients of determination of GEI variances, the genotypes raking varies more along the environmental gradient.

4. “Finally, I would emphasize that ‘less is more’ in this case. The more clearly defined the manuscript becomes, the higher the impact it will have in its field.”

“Minor comment: - The colors in most figures are hard to read and follow.”

Response: We re-made the Results and Discuss almost entirely. (See Results and Discussion)

Thank you very much for the review and for the excellent contributions to improvement of this manuscript.

Alves, R. S., Resende, M. D. V., Azevedo, C. F., Silva, F. F., Rocha, J. R. do A. S. de C., Nunes, A. C. P., Carneiro, A. P. S., & Santos, G. A. (2020). Optimization of Eucalyptus breeding through random regression models allowing for reaction norms in response to environmental gradients. *Tree Genetics & Genomes*, *16*(2), 38. https://doi.org/10.1007/s11295-020-01431-5

Brewer, M. J., Butler, A., & Cooksley, S. L. (2016). The relative performance of AIC, AIC C and BIC in the presence of unobserved heterogeneity. *Methods in Ecology and Evolution*, *7*(6), 679–692. https://doi.org/10.1111/2041-210X.12541

Burnham, K. P., & Anderson, D. R. (2004). Multimodel inference: understanding AIC and BIC in model selection. *Sociological Methods and Research*, *33*, 261–304.

Cavanaugh, J. E., & Neath, A. A. (2019). The Akaike information criterion : Background , derivation , properties , application , interpretation , and refinements. *Wiley Computational Statistics*, *11*(January), 1–11. https://doi.org/10.1002/wics.1460

Gianola, D., & Fernando, R. L. (1986). Bayesian methods in animal breeding theory. *Journal of Animal Science*, *63*(1), 217–244.

Melo, V. L. de, Marçal, T. de S., Rocha, J. R. A. S. de C., dos Anjos, R. S. R., Carneiro, P. C. S., & Carneiro, J. E. de S. (2020). Modeling (co)variance structures for genetic and non-genetic effects in the selection of common bean progenies. *Euphytica*, *216*(5), 77. https://doi.org/10.1007/s10681-020-02607-9

Neath, A. A., & Cavanaugh, J. E. (2012). The Bayesian information criterion: background, derivation, and applications. *Wiley Interdisciplinary Reviews: Computational Statistics*, *4*(2), 199–203. https://doi.org/10.1002/wics.199

Resende, M. D. V. De, & Duarte, J. B. (2007). Precisão e controle de qualidade em experimentos de avaliação de cultivares. *Pesquisa Agropecuária Tropical*, *37*(3), 182–194. https://doi.org/10.5216/pat.v37i3.1867

Van Eeuwijk, F. A., Bustos-Korts, D. V., & Malosetti, M. (2016). What should students in plant breeding know about the statistical aspects of genotype × Environment interactions? *Crop Science*, *56*(5), 2119–2140. https://doi.org/10.2135/cropsci2015.06.0375

Verbyla, A. P. (2019). A note on model selection using information criteria for general linear models estimated using REML. *Australian and New Zealand Journal of Statistics*, *61*(1), 39–50. https://doi.org/10.1111/anzs.12254
