# Supplementary material for: Multiple-trait, random regression, and compound symmetry models for analyzing multi-environment trials in maize breeding
Source: PLoS One. 2020 Nov 20;15(11):e0242705. doi: 10.1371/journal.pone.0242705 (PMC7678961; doi:10.1371/journal.pone.0242705)
Supplement: Attachment — Submitted filename: Response Letter_IFC.docx [file pone.0242705.s006.docx]

**Response letter**

Authors did not implement most of the changed they could in this revised version. I still believe this paper could have a big impact, but the writing has to improve. Your goal is to compare methods to infer GE, then provide practical guidance to users so they can not only choose the most adequate method but discuss their limitations and biological interpretation of model parameters. I see you replied to my concern about parameter uncertainty in the rebuttal letter, but I haven't seen any sentence about it in the actual manuscript. This is a real concern and most readers will think about it when they read your paper. So even if you're not willing to implement any Bayesian technique, at least talk about the limitations of your protocol to compare methods.

We agree, and we made several changes/corrections in the manuscript. The aim of this study was to compare several statistical models in the Fisherian context (REML/BLUP procedure) for MET analysis in maize breeding. Currently, REML/BLUP is the standard procedure for estimation of variance components and optimal selection in plant breeding (Resende 2016). We included one paragraph highlighting the possibilities to fit the same models through Bayesian inference or through Hierarchical Generalized BLUP (HG-BLUP). Please see the text highlight in blue in the manuscript.

Thank you very much for the review and for the excellent contributions to improvement of this manuscript.

**Reviewer #1**:

1. “You still need to provide the difference between the best model and the other models in your model selection table. This is the deltaAIC. If you include an additional column with dAIC you don't need to indicate the best model using #, which is kind of weird. Notice that the criteria to select models in the information theoretical framework is not the raw AIC value per se (since the AIC is dimensionless, because it's derived from the log likelihood), but the difference in AIC between competing models (see Burnham & Anderson p. 70-2). Usually a difference in AIC higher than 2 demonstrates a unequivocal support for the model with the lowest AIC.”

We agree, and we described the AIC and the Δ_AIC_ in the Material and Methods section. Also, we included a column of Δ_AIC_ in Table 1 and we highlight the importance of the Δ_AIC_ in the selection of models in the Discussion section. Please see the text highlight in blue in the manuscript.

2. “The discussion still contains sentences about AIC and LRT, which to me should be removed. Also, some sentences of the discussion repeats parts of the methods, which doesn't make sense. Discussion is still very lengthy to the amount of results you have and must be shortened. See my comments in the pdf attached. Avoid citing tables in the discussion.

We agree, and we removed the sentences about AIC and LRT in the discussion section. We removed the citations of Tables in the discussion section. Also, we re-written almost everything in the discussion section, trying to be clearer and objective. Please see the text highlight in blue in the manuscript.

3. (line 302-304) “and then what? What's the implication of this result? You're simply restating the result here in the discussion, when you should be indeed **explaining** them... Also, there's no citation in this paragraph...”

We re-written the discussion section. At this point we include a topic of variance components and genetic parameters. We discuss deeper about these parameters and their importance in maize breeding. Please see the text highlight in blue in the manuscript.

Thank you very much for the review and for the excellent contributions to improvement of this manuscript.

**Reviewer #2:**

“I have now reviewed the revised version of Coelho et al, in which the authors present a detailed multi-environmental trial analysis of 84 maize hybrids. As mentioned in my previous review, the goal of the paper is clear. The manuscript clearly aims to better understand the extent to which GEI interactions might be relevant to maize breeding programs. The approach is also sound. The authors use a simple model-fitting approach and directly compare the model fit of three main model classes (CSM, MTM and RRM).

Having said that, I have now read this paper multiple times and have postponed writing my review to gather my thoughts. As a reviewer, I strive to make suggestions that will allow authors to improve their manuscript. My sincere hope is that the revised version will be clearer and make a stronger argument in favor of the manuscript. In my view, the revised version of this manuscript does not succeed in addressing most of the comments raised by the editor and me in its previous iteration.”

1. “I still find the manuscript to have significant shortcomings in regard to organization/clarity and in placing the work in a greater context.”

We agree, and we made several changes/corrections in the manuscript. Please see the text highlight in blue in the manuscript.

2. “As I mentioned before, the goal of the manuscript is straightforward: compare these three model classes and infer the practical consequences of choosing one over the other (in terms of GEI). While the manuscript presents in detail the difference between these models in terms of their estimated components, it spends little to no time exploring the practical consequences of choosing one over the other.

2.1) “Yes, the parameters obtained by the different models are different, but what does that mean in practical terms?”

In practical terms, the best fitted model can provide more accurate estimates of genetic parameters and predicted genetic values, and consequently can maximize the efficacy of the breeding program. This is the first study that apply the RRM for analysis of MET in maize breeding and demonstrated the great advantage of this models in MET analysis.

2.2) “To what degree will it change the standard agricultural practice if the genotype rankings are so similar and if the metrics of selective accuracy are nearly identical between at least two of the models?”

The main advantage of RRM over the CSM and MTM is the ability to predict genotypic performance in environments where a genotype has not been evaluated (and this can increase the efficacy of breeding programs). Besides that, MTM tends to present problems in relation to convergence due to the large number of parameters estimated. Thus, the MTM became prohibitive when the number of environments is high.

2.3) “Also, as mentioned by the AE, what are the uncertainties around these parameters? Uncertainty in parameters is essential for a proper interpretation of the results. Should we observe a similar type of discrepancy between the three models if we were working with another population in different environments? In other words, the manuscript needs to go beyond reporting the technical details and expand on the biological implications.”

We added in Table 2 the standard errors of the variance component estimates. The results for another population evaluated in other environments can be different, but the statistical properties of the models will remain unchanged.

2.4) “As admitted in the main text of the manuscript, some of these models do not even have parameters that are capable of estimating GEI components directly (such as the MTM), despite that being the main goal of the manuscript.”

The MTM are the most complete models to deal with GEI. The fact that these models don’t be able to estimate the variance of the GEI interaction is not a limitation, because the GEI can be observed through the genotype ranking. Please see the text highlight in blue in the manuscript.

3) “Another issue that was not properly addressed was the use of figures.”

3.1) “Other than the addition of few red lines in one of the figures (Figure 1), there was no noticeable attempt to make the figures clearer. Why not highlight the crossing interactions (if that is the main argument being made) in Figure 1?”

Aiming to clarify the Fig 1, we split in A and B. In B, we reduced the number of reaction norms to make it easier to see the reaction norms and their intersections (what represent the presence of the GEI).

3.2) “Also, the figures came without caption this time. The figure with large matrices (which has no number) is very hard to read.”

We made some changes in the letter’s font, and we changed it to bold letter, to become it easier to read.

In short, the revised version of Coelho et al. remains a potentially interesting contribution to the field but falls short of making a stronger case at this point in time.

Thank you very much for the review and for the excellent contributions to improvement of this manuscript.
